# The oxidation of dehydroascorbic acid and 2,3-diketogulonate by distinct reactive oxygen species

**DOI:** 10.1042/BCJ20180688

**Published:** 2018-11-09

**Authors:** Rebecca A. Dewhirst, Stephen C. Fry

**Affiliations:** The Edinburgh Cell Wall Group, Institute of Molecular Plant Sciences, The University of Edinburgh, The King's Buildings, Edinburgh EH9 3BF, U.K.

**Keywords:** ascorbic acid metabolism, dehydroascorbic acid, diketogulonate, reactive oxygen species, singlet oxygen, superoxide

## Abstract

l-Ascorbate, dehydro-l-ascorbic acid (DHA), and 2,3-diketo-l-gulonate (DKG) can all quench reactive oxygen species (ROS) in plants and animals. The vitamin C oxidation products thereby formed are investigated here. DHA and DKG were incubated aerobically at pH 4.7 with peroxide (H_2_O_2_), ‘superoxide’ (a ∼50 : 50 mixture of ${\text{O}}_{2}^{\cdot -}$ and $\text{H}{\text{O}}_{2}^{\cdot }$), hydroxyl radicals (^•^OH, formed in Fenton mixtures), and illuminated riboflavin (generating singlet oxygen, ^1^O_2_). Products were monitored electrophoretically. **DHA** quenched H_2_O_2_ far more effectively than superoxide, but the main products in both cases were 4-*O*-oxalyl-l-threonate (4-OxT) and smaller amounts of 3-OxT and OxA + threonate. H_2_O_2_, but not superoxide, also yielded cyclic-OxT. Dilute Fenton mixture almost completely oxidised a 50-fold excess of DHA, indicating that it generated oxidant(s) greatly exceeding the theoretical ^•^OH yield; it yielded oxalate, threonate, and OxT. ^1^O_2_ had no effect on DHA. **DKG** was oxidatively decarboxylated by H_2_O_2_, Fenton mixture, and ^1^O_2_, forming a newly characterised product, 2-oxo-l-*threo*-pentonate (OTP; ‘2-keto-l-xylonate’). Superoxide yielded negligible OTP. Prolonged H_2_O_2_ treatment oxidatively decarboxylated OTP to threonate. Oxidation of DKG by H_2_O_2_, Fenton mixture, or ^1^O_2_ also gave traces of 4-OxT but no detectable 3-OxT or cyclic-OxT. In conclusion, DHA and DKG yield different oxidation products when attacked by different ROS. DHA is more readily oxidised by H_2_O_2_ and superoxide; DKG more readily by ^1^O_2_. The diverse products are potential signals, enabling organisms to respond appropriately to diverse stresses. Also, the reaction-product ‘fingerprints’ are analytically useful, indicating which ROS are acting *in vivo*.

## Introduction

Ascorbate (AA; one form of vitamin C) is the major low-molecular-weight, water-soluble antioxidant in plants and animals, acting to quench reactive oxygen species (ROS). It is present in all metabolically active cell types and sub-cellular compartments, and some AA is released into blood plasma [1] and the plant apoplast (solution which permeates the cell wall) [2–6]. The major biosynthetic pathways of AA in animals [7] and plants [8] are well characterised, but its degradation pathways have yet to be fully elucidated. One reason why the oxidative degradation of AA, and of its own downstream products, by ROS is important is that these reactions ‘quench’ these potentially damaging ROS. Other reasons are that the biosynthesis : degradation ratio dictates steady-state AA levels *in vivo*, e.g. in fruits and vegetables, and that degradation contributes to vitamin losses during food storage [9] and cooking [10].

**Figure 1.**
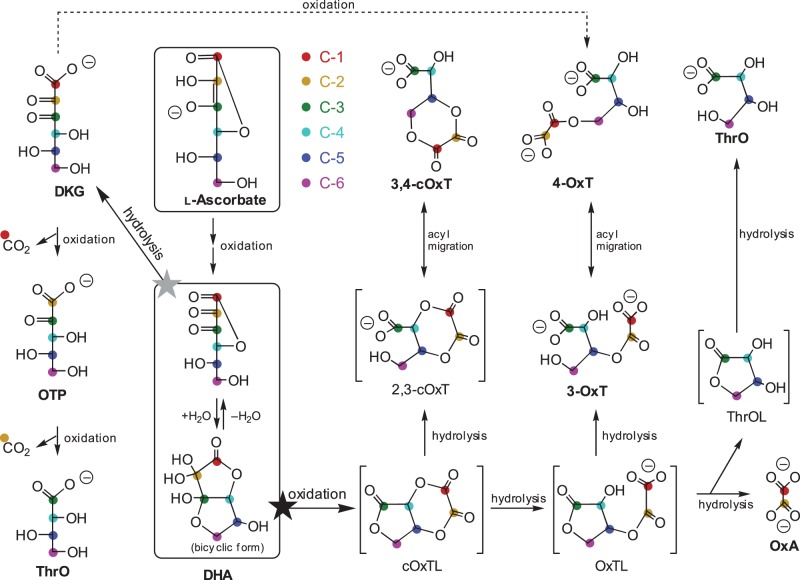
Catabolism of AA. All the reactions shown can occur non-enzymically; some are also promoted by plant enzymes. The two-electron oxidation of AA proceeds via AA-free radical (not shown), yielding DHA. This unionised product, which mainly exists in the bicyclic form, is at a metabolic branch-point: it can be either hydrolysed (grey star) to DKG or oxidised (black star) to an unstable intermediate which is proposed to be cOxTL. The structures of products formed from both DKG and cOxTL by various ROS (in steps labelled ‘oxidation’) are shown. Note that ThrO and 4-OxT can arise by at least two routes. Compounds sufficiently stable to be detected after electrophoresis are labelled in bold; hypothetical intermediates are shown in square brackets. The probable derivation of carbon atoms, referred to the original AA molecule, is indicated by coloured dots; however, it is not possible to be definitive about the disposition of former C-1 and C-2 within OxT and cOxT compounds. The pathways are from Parsons et al. [17], updated with the OTP branch (present work).

AA is unstable in aqueous solutions under aerobic conditions, the first oxidation product being AA free radical, which can be either reduced back to AA *in vivo* or oxidised further to produce dehydro-l-ascorbic acid (DHA; Figure 1) [11]. The oxidation of AA to DHA is reversible *in vivo*, partly owing to dehydroascorbic acid reductase action [12]. However, DHA represents a branch-point in AA catabolism and can undergo further irreversible degradation (Figure 1), resulting in a permanent loss of vitamin C. DHA can be hydrolysed to 2,3-diketo-l-gulonate (DKG) or oxidised to a range of products such as l-threonic acid (ThrO), oxalic acid (OxA), and their esters [13–16]. Investigations of the fate of DHA under oxidising conditions *in vitro* revealed the probable existence of a short-lived, highly reactive intermediate (proposed to be cyclic-2,3-oxalyl-l-threonolactone; cOxTL), which simultaneously forms three major end-products (Figure 1): cyclic oxalyl threonate (cOxT), oxalyl threonate (OxT), and OxA (plus ThrO), in a ∼6 : 1 : 1 ratio [17,18]. OxT exists as at least two isomers, 3-OxT and 4-OxT [17], of which 4-OxT is the more stable [14]. Interconversion between these isomers occurs *in vivo* [17] and *in vitro* [18]. In the presence of esterases *in vivo*, and during prolonged enzyme-free incubations *in vitro*, irreversible hydrolysis of some of the initially formed products can also occur (cOxT → OxT → OxA + ThrO; Figure 1) [14,17,19]. Also, transacylase activities can transfer the oxalyl group from OxT to acceptor substrates, e.g. carbohydrates [20].

If oxidation is limited by exclusion of ROS, DHA in aqueous solution predominantly undergoes hydrolysis to form DKG (Figure 1). This hydrolysis is probably irreversible *in vivo* [12,21]. DKG itself can be oxidised into an unknown compound (‘H’), which itself can be further oxidised to ThrO; therefore, DKG can, like AA and DHA, act as an antioxidant [17,18]. Compound ‘H’ has been characterised in the present work and is indicated in Figure 1 as OTP (2-oxo-l-*threo*-pentonate). Lactonised products of DKG have also been reported, including simple lactones [22] branched-chain rearrangement products [17] and a reduction product [23].

All the oxidation reactions mentioned require electron acceptors such as ROS. The major biological ROS are hydrogen peroxide (H_2_O_2_), superoxide (here taken to include ${\text{O}}_{2}^{\cdot -}$ and its unionised form $\text{H}{\text{O}}_{2}^{\cdot }$), hydroxyl radicals (^•^OH), and singlet oxygen (^1^O_2_). Although often viewed as detrimental to cellular survival, ROS are naturally present in healthy organisms and are generated during normal aerobic metabolism, e.g. in mitochondria, chloroplasts, peroxisomes, and the plant cell wall. For instance, superoxide is produced in mitochondria by the one-electron reduction of O_2_ [24,25], and at cell surfaces by NADPH oxidases [26]. Superoxide is also generated in organisms treated with the herbicide paraquat [27,28]. ROS levels are often elevated *in vivo* in response to abiotic stresses (e.g. ultraviolet radiation [29], drought [30], mineral deficiency, heavy metal [31] or boron toxicity, salinity [30], and ozone pollution [32]), and microbial infection [33]. H_2_O_2_, superoxide, and ^•^OH can all be produced in the chloroplast [34–36]. ^1^O_2_ can be produced in biologically relevant situations by exposure to ultraviolet (e.g. in the presence of tryptophan; [37,38]) and visible light (e.g. in the presence of riboflavin; [39,40]), and has been reported to be responsible for the majority of photo-oxidative damage in leaves [41]. ^•^OH is the most potent but also the shortest-lived ROS [42,43].

Some ROS — generated at the right time and place — have beneficial biological roles [44], e.g. H_2_O_2_ and ^1^O_2_ as signalling molecules [45–49]; superoxide made during the oxidative burst as a defence against pathogens [50–52]; H_2_O_2_ as a reactant to synthesise lignin [53,54] and to cross-link proteins and feruloyl-polysaccharides in the cell wall, thus strengthening the wall and preventing pathogen ingress [44,55,56]; and ^•^OH as a wall-loosening agent enabling cell expansion and fruit softening [57–60].

On the other hand, excess ROS can damage cells and are widely implicated in ageing and diseases such as atherosclerosis [61,62]. For example, superoxide can attack membranes, especially their unsaturated fatty acid residues; ^•^OH can react with DNA, proteins, and lipids, causing mutation, denaturation, and membrane permeabilisation, respectively; and ^1^O_2_ can inactivate enzymes, including xyloglucan endotransglucosylase/hydrolases [40]. Quenching of ROS may therefore often be beneficial and is potentially achieved by AA, DHA, and DKG. However, since ROS are sometimes beneficial, their scavenging is not always advantageous to the organism, and the antioxidant pathways leading to scavenging are complex and need to be tightly controlled.

AA can react directly with ^•^OH, superoxide, and ^1^O_2_, and can reduce H_2_O_2_ via the ascorbate–glutathione pathway, mitigating oxidative stress [63,64]. For example, increased apoplastic AA concentrations correlate with increased tolerance of oxidative stresses such as ozone [5,65,66], and apoplastic AA represents a cell's first line of defence against ozone [67–69]. In addition, AA quenches ^1^O_2_, e.g. in food systems [70] and in plants [71,72]. However, the products formed from the reaction of AA and DHA with ^1^O_2_ have not been defined.

The actions of the various ROS are interconnected, with many ROS degrading to form H_2_O_2_. Likewise, ozone in aqueous solution can form ^•^OH [73] and ^1^O_2_ [74]. The aim of this study was therefore to further elucidate the pathways of AA catabolism, via the action of various ROS on DHA and DKG, and to distinguish the oxidation products generated by different ROS. The outcome defines breakdown-product ‘fingerprints’ enabling us to recognise which ROS were being scavenged by DHA or DKG, and suggesting mechanisms by which organisms may recognise which ROS are prevalent and activate appropriate resistance responses.

## Materials and methods

All chemicals used were purchased from Sigma–Aldrich (Poole, U.K.) or Fisher Chemicals (Loughborough, U.K.). l-[1-^14^C]Ascorbic acid was purchased from Amersham Pharmacia Biotech UK Ltd.

### Electrophoresis

Aqueous samples were loaded onto Whatman No. 3 paper and electrophoresed at 2.5–3.5 kV for 30–70 min in a buffer of pH 2 (formic acid/acetic acid/water, 1 : 4 : 45, v/v/v) or pH 6.5 (pyridine/acetic acid/water, 33 : 1 : 300, v/v/v) [75].

Orange G (2 µl, 10 mM) was added to all samples as an internal marker, and electrophoretic mobilities (*m*_OG_) are calculated relative to orange G. Neutral compounds move a small distance away from the origin owing to electro-endo-osmosis, so mobilities were calculated with a neutral marker (e.g. DHA) as *m*_OG_ = 0. After long electrophoresis runs, orange G ran off the paper, and *m*_ThrR_ [mobility relative to threarate (*m*_ThrR_ = 1.0) and glucose (*m*_ThrR_ = 0.0)] was used instead of *m*_OG_. Ascorbate-related compounds were stained with AgNO_3_ [76]. Paper electrophoretograms containing ^14^C-labelled compounds were exposed to photography film (Kodak BioMax MR-1 film) for 7 days.

### Purification of [^14^C]DHA by anion-exchange column chromatography

[1-^14^C]DHA was obtained from 100 µM [1-^14^C]AA treated with AA oxidase (from *Cucurbita* species, 1 U µl^–1^) in 10 mM formate (pyridinium, pH 5), for 30 min. The solution was then passed through a 50-µl bed volume Dowex 1 anion-exchange column that had previously been washed in 500 µl each of, sequentially, (a) 0.5 M NaOH, (b) 0.5 M formic acid, (c) 2 M sodium formate and (d) 10 mM formate (pyridinium, pH 5.0) buffer. The [^14^C]DHA, which had no affinity for the column, was eluted in H_2_O.

### Preparation of diketogulonate

Usually, DKG was produced by hydrolysis of DHA. A solution of 3 M DHA in DMF (40 µl) was mixed with 200 µl of 0.75 M NaOH and incubated for 30 s. Acetic acid (1.5 M, 200 µl) was added to stop the reaction. The DKG was diluted to 50 mM in H_2_O (thus 62.5 mM sodium) and stored at −80°C.

The DKG used in some experiments (specified in legends) was prepared by iodate treatment of AA. A solution containing AA (0.12 M) and potassium iodate (0.36 M) was incubated for 5 min. KOH (1 M) was then added dropwise until the solution became colourless. Cold ethanol (8 volumes, −20°C) was added, precipitating the DKG as its K^+^ salt. The precipitated DKG was vacuum filtered, rinsed in 70% ethanol, dried, and stored at −80°C.

### ROS reactions

All ROS reactions were carried out at ∼20°C in 0.1–0.2 M acetate (Na^+^) buffer, pH 4.7 or 4.8 over the time-courses indicated in Results. Products were stored at −80°C before electrophoresis.

Commercial H_2_O_2_ was used. Reactions with H_2_O_2_ were stopped at the desired time-point by the addition of catalase (bovine liver) to 2.5 mg/ml.

Superoxide was added as commercial KO_2_. It is impossible to prepare a stock solution of aqueous KO_2_ owing to its short half-life. Instead, we prepared a 1.0 M suspension of KO_2_ powder in dry hexane and added 0–0.075 volume of the rapidly stirring suspension to 1 volume of a buffered aqueous solution of DHA or DKG; vigorous shaking was continued until all the KO_2_ had dissolved, theoretically giving an initial superoxide concentration of 0–75 mM. Each mole of KO_2_ will rapidly yield 1 mol of KOH (regardless of whether it underwent dismutation and/or reacted with DHA or DKG). To minimise the resulting rise in pH, we included 200 mM acetate buffer, pH 4.7; the highest concentration of KO_2_ tested (75 mM) will have raised the pH of this to ∼5.5, which is unlikely to have had any appreciable effect on the fate of AA derivatives. The buffer used for superoxide incubations contained catalase (2.5 mg/ml), preventing oxidation of DHA or DKG by the H_2_O_2_ formed from superoxide by dismutation. Stopping superoxide reactions was not necessary because this radical has an extremely short half-life, and we had included catalase to scavenge the H_2_O_2_ by-product.

We produced the hydroxyl radical with an equimolar mixture of FeSO_4_, H_2_O_2_, and EDTA (ethylenediaminetetraacetate) (each 1, 10, or 50 mM). Any ^•^OH action was stopped at selected time-points by the addition of ethanol to 50% (v/v).

Singlet oxygen was produced from 1 mM riboflavin (or 10 mM for Supplementary Figure S7) in a glass tube placed 40 cm from a fluorescent lamp. The reaction was stopped by shading and freezing.

## Results

### Identification of two previously unknown DKG metabolites

Treatment of DKG with H_2_O_2_ yielded at least five products: CPA [(formerly called compound **E**) proposed to be 2-carboxy-l-*threo*-pentonate (i.e. ‘2-carboxy-l-xylonate’ or ‘2-carboxy-l-lyxonate’, which are synonyms)], CPL [(formerly called compound **C**) proposed to be a mixture of 2-carboxy-l-*threo*-pentonolactones (2-carboxy-l-xylonolactone plus 2-carboxy-l-lyxonolactone)], an unidentified compound (‘**H**’ of Parsons & Fry [18]), threonate, and a product that electrophoresed in the OxT zone (but whose identity required checking since OxT had not previously been noted as a DKG product) (Figure 4; discussed in more detail in the following section). In the pathway, DKG → **H** → ThrO, both steps require the presence of ROS [18,77]. CPA and CPL were provisionally identified as non-oxidative by-products (Figure 6 of ref. [17]): CPA as 2-carboxy-l-*threo*-pentonate, formerly called compound E, and CPL as 2-carboxy-l-*threo*-pentonolactones, formerly compound C [17]. These will be further characterised in a future manuscript. Therefore, in the present work, we investigated the chemistry of the two DKG oxidation products: **H** and the putative OxT.

#### Compound **H** is 2-oxo-l-*threo*-pentonate (‘2-keto-l-xylonate’)

**Figure 2.**
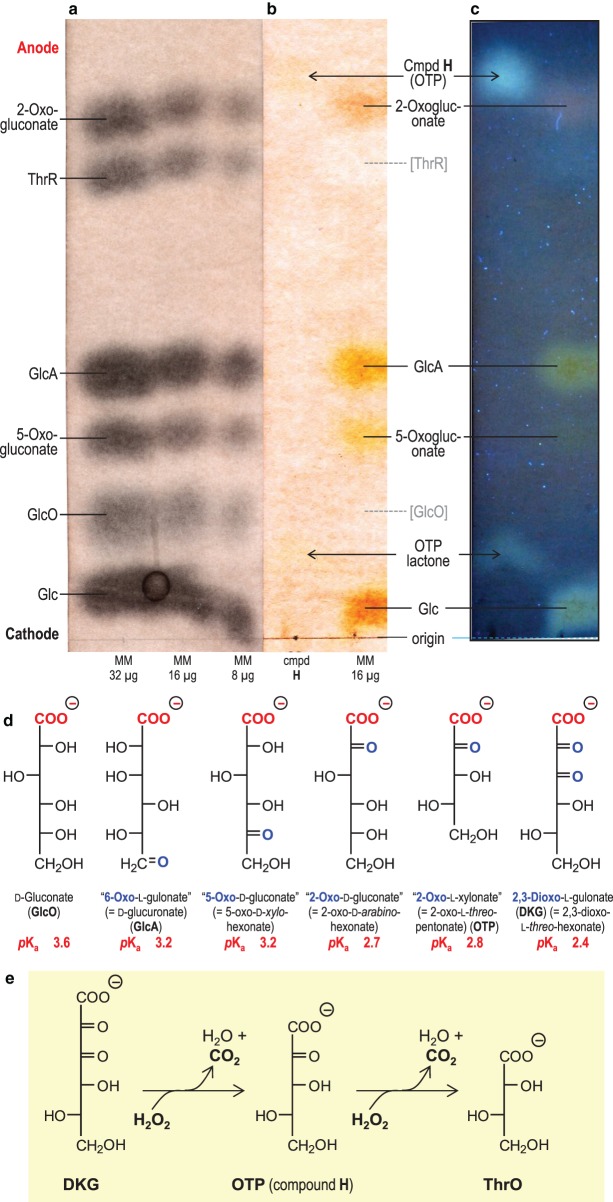
Compound H is a 2-oxo-aldonic acid. (**a**–**c**) Electrophoresis at pH 2.0 and 3.0 kV for 3.5 h. The sample containing compound **H** in (**b**) was 20 µl of the 5-h reaction products between DKG and H_2_O_2_ (see Figure 4). MM, marker mixtures containing 8, 16, or 32 µg, each, of glucose (Glc), gluconic acid (GlcO), 2-oxogluconic acid, 5-oxogluconic acid, glucuronic acid (GlcA), and threaric acid (ThrR; l-tartaric acid). Sections of the electrophoretogram were stained with (**a**) AgNO_3_, revealing all sugar-like compounds, or (**b**,**c**) aniline hydrogen-phthalate, revealing only reducing sugars (not GlcO or ThrR). (**c**) shows part (**b**) as seen under a 360-nm UV lamp, revealing the fluorescence of reducing sugars. [A high NaOAc buffer content of the compound **H** sample has distorted the position of neutral substances in this and neighbouring samples.] (**d**) Fischer projection formulae with synonyms and approximate p*K*_a_ values. (**e**) The deduced oxidative decarboxylation pathway occurring when H_2_O_2_ acts on DKG.

Oxidation of DKG with H_2_O_2_ yielded CO_2_, detected by gas chromatography, suggesting that **H** might be a C_5_ compound [78]. By mass spectrometry, Deutsch [77] found that **H** has the correct mass to be an oxo-pentonate [i.e. a 3,4,5-trihydroxy-2-ketopentanoate; C_5_H_8_O_6_ (in the unionised form)], which could be formed from DKG (C_6_H_8_O_7_ in the unionised form) thus:

$${\text{C}}_{6}{\text{H}}_{8}{\text{O}}_{7}+[\text{O}]\to {\text{C}}_{5}{\text{H}}_{8}{\text{O}}_{6}+\text{C}{\text{O}}_{2},$$

where [O] is an oxygen atom from H_2_O_2_ (to simplify balancing, we present all equations with the compounds in their unionised form). The theoretical oxidative decarboxylation product of DKG is OTP (synonyms: 2-oxo-l-xylonate, 2-oxo-l-lyxonate, 2-keto-l-xylonate, l-xylosonate; Figures 1 and 2e), our proposed identity for **H**.

No authentic OTP was available to test this identity; however, the chemically similar 2-oxo-*arabino*-hexonate (OAH; ‘2-keto-gluconate’) was tested by electrophoresis. OAH is a much stronger acid than its parent compound, gluconate, with p*K*_a_ values ≈ 2.7 [79] and 3.9 [80], respectively. Electrophoresis at pH 2.0 (Figure 2) showed that compound **H** and OAH had similar, high, mobilities, indicating that they share an unusually strong acidity. Accurate mobilities can be read from Supplementary Figure S1. By applying Offord's rules [75,81], we estimate the p*K*_a_ of compound **H** to be ∼2.8 (Supplementary Figure S2).

Figure 2d shows a simple aldonic acid (gluconate; GlcO) alongside several oxo-aldonic acids. Increasing proximity of neutral C=O groups to the carboxylate group (–COO^−^) renders the latter more acidic (lower p*K*_a_; Figure 2a; faster-migrating on electrophoresis at pH 2). The strong acidity of compound **H** thus supports its proposed identity as a 2-oxo-pentonate.

Staining reactions supported the proposal that **H** is an oxo-aldonate. It was stainable with AgNO_3_ (Supplementary Figure S1), like all sugar acids. In addition, it stained with aniline hydrogen-phthalate (Figure 2b,c), which detects reducing sugars (aldoses and ketoses; [76]). The reducing nature of **H**, attributable to an oxo group, is a property it shares with glucose, 2-oxogluconate, 5-oxogluconate, and glucuronate (‘6-oxo-l-gulonate’), but not gluconate or threarate (Figure 2). Spots produced by aniline hydrogen-phthalate often exhibit a characteristic fluorescence under ultraviolet, and indeed, **H** showed a strong bluish fluorescence under 360-nm UV (Figure 2c and Supplementary Figure S3). The faint spot accompanying **H** and running near the origin (Figure 2c; position slightly distorted by the heavy loading of sodium acetate) has the same colour and fluorescence properties as **H**, and is probably a lactone of OTP.

#### OxT is a minor ROS product of DKG

OxT had not been reported as a DKG oxidation product, though well established as a DHA catabolite. Therefore, putative OxT spots produced by ROS treatment of DHA or DKG were compared after elution from an electrophoretogram and treatment with or without cold alkali, which quickly hydrolyses OxT to ThrO + OxA. We confirmed that the ‘OxT’ spot, whether produced from DHA or from DKG, and whether by H_2_O_2_ or by superoxide, completely disappeared upon alkali treatment, yielding ThrO + OxA (Supplementary Figure S3c). Thus, OxT is indeed a minor product formed by ROS treatment of DKG.

### The reactions of DHA and DKG with H_2_O_2_ and superoxide

**Figure 3.**
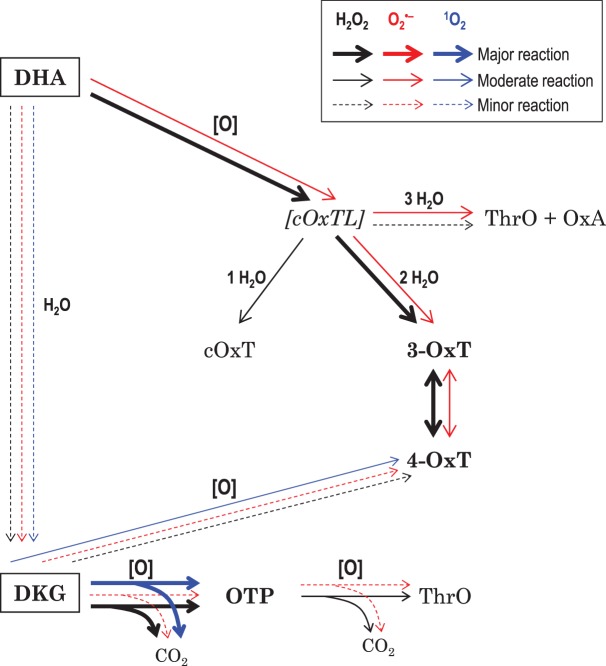
Deduced pathways of dehydroascorbic acid and diketogulonate degradation in the presence of H_2_O_2_, superoxide, or singlet oxygen. The two substrates tested are in boxes. **Black**, **red,** and **blue** arrows show the H_2_O_2_, superoxide, and singlet oxygen pathways, respectively. Arrow thickness indicates the prevalence of reaction. ‘H_2_O’ indicates a hydrolysis reaction; ‘[O]’ indicates an oxidation reaction (caused by ROS). *[cOxTL]* is a hypothetical highly reactive intermediate. For abbreviations, see Figure 1.

The products of oxidation and/or hydrolysis formed when DHA and DKG are subjected to treatment with H_2_O_2_ and superoxide are summarised (Table 1), and the deduced pathways are presented (Figure 3). The evidence is given in the following paragraphs.

**Table 1:** Major and minor products formed by ROS action on DHA and DKG

Sub­strates and products	Loss of substrates and formation of products during reaction of							
	DHA + H_2_O_2_	DKG + H_2_O_2_	DHA **+** superoxide	DKG **+** superoxide	DHA + ^1^O_2_	DKG + ^1^O_2_	DHA + Fenton mix^†^	DKG + Fenton mix^†^
DHA	↓*t*_1/2_ ≈ 2.5 h	*n/a*	*slight loss*	*n/a*	↓*t*_1/2_ ≈ 24 h	*n/a*	↓*t*_1/2_ < 6 s	*n/a*
DKG	(↓*)	↓*t*_1/2_ ≈ 2.5 h	–*	*slight loss*	(↑*)	↓*t*_1/2_ ≈ 8–24 h	–*	↓*t*_1/2 _< 6 s
3-OxT	++++	–	+	–	–	–	+++ (nr)	–
4-OxT	++++	+	++	+	+	+		+
cOxT	++	–	–	–	–	–	–	–
OxA	±	–*	+	–*	?	?	++++	?
OTP	(+)	+++	–	+	(++)	++	–	++
ThrO	(+)	++	±	+	–	–	++	++
CPA	–	–*	–	–*	–	↑*	–	++
CPL	–	–*	–	–*	–	↑*	–	↑*

↓: Loss of substrate in the presence of ROS (*t*_1/2_ = approximate half-life).

–* Present as a contaminant in the substrate; no change during ROS treatment.

↓* Present as a contaminant in the substrate; decreasing during ROS treatment.

↑* Present as a contaminant in the substrate; increasing during ROS treatment.

*slight loss*: little observable loss of substrate during ROS treatment.

*n/a*, not applicable (DHA is not produced from DKG).

–, Absent.

±, +, ++, +++, ++++, Increasing during ROS treatment (±, trace product; ++++, major product).

( ), i.e. entries in parentheses, Product of the contaminating DKG, not a direct product of DHA.

nr, Isomers (3-OxT and 4-OxT) not resolved.

^†^Nature of the responsible ROS not identified.

#### The major products of H_2_O_2_ action on DHA are OxT isomers

**Figure 4.**
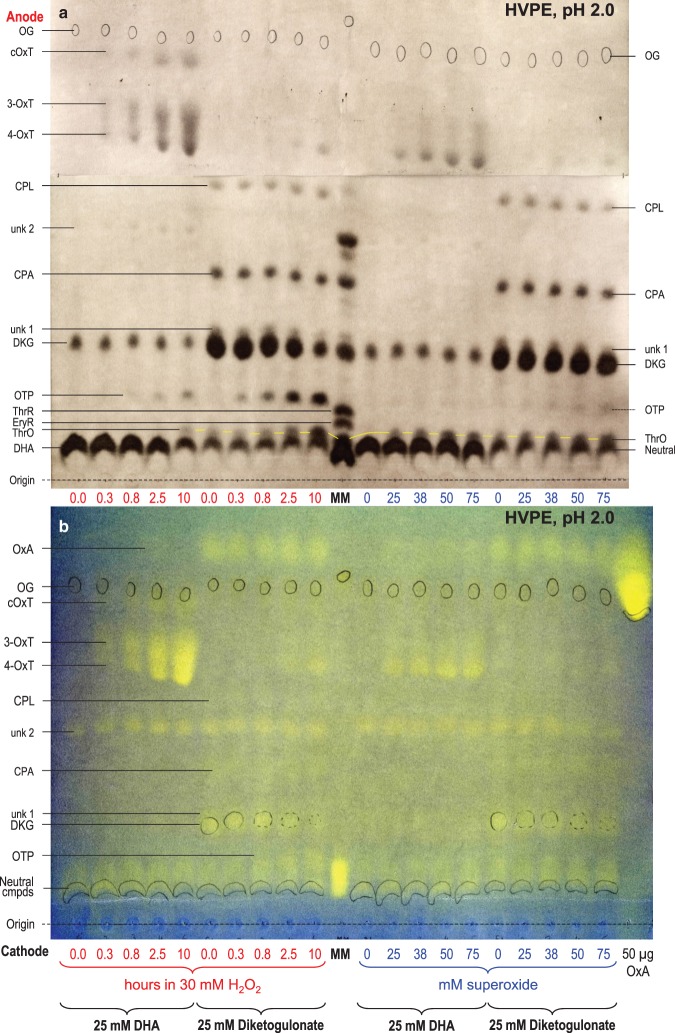
Reaction of dehydroascorbic acid and diketogulonate with H_2_O_2_ and superoxide: electrophoresis at pH 2.0. DHA or DKG (25 mM) was incubated with 30 mM H_2_O_2_ for 0–10 h (left half of each electrophoretogram), or with 0–75 mM KO_2_ in the presence of catalase for a few seconds (right half). All reaction mixtures were buffered with 200 mM acetate (Na^+^), pH 4.7. Samples were electrophoresed for 60 min at pH 2.0. (**a**) 10 µl sample run at 2.5 kV with AgNO_3_ staining. (**b**) 20 µl sample run at 2.0 kV [so that the fast-migrating OxA would be retained on the paper] with bromophenol blue staining. The ‘0.0-hour’ time-points represent DHA or DKG with no H_2_O_2_. Each loaded sample also contained 2.8 nmol of Orange G (OG) as an internal marker (circled in pencil). In (**a**), the marker mixture (MM) contained glucose (neutral, co-migrating with DHA), ThrO, EryR, ThrR, and various AA catabolites. In (**b**), MM contained glucose, ThrO, EryR, and ThrR. Oxalic acid (50 µg) was run as a separate marker. Abbreviations: cOxT, cyclic oxalyl-l-threonate; CPA, 2-carboxy-l-*threo*-pentonate, formerly called compound **E** (Parsons et al. [17]); CPL, 2-carboxy-l-*threo*-pentonolactone, formerly called compound **C** (probably two epimers; Parsons et al. [17]); DHA, dehydro-l-ascorbic acid; DKG, 2,3-diketo-l-gulonate; EryR, erythrarate (*meso*-tartrate); OTP, 2-oxo-*threo*-pentonate (compound **H**); OxT, 3- and/or 4-*O*-oxalyl-l-threonate; ThrO, l-threonate; ThrR, l-threarate (l-tartrate). The ThrO spots are joined by yellow lines.

**Figure 5.**
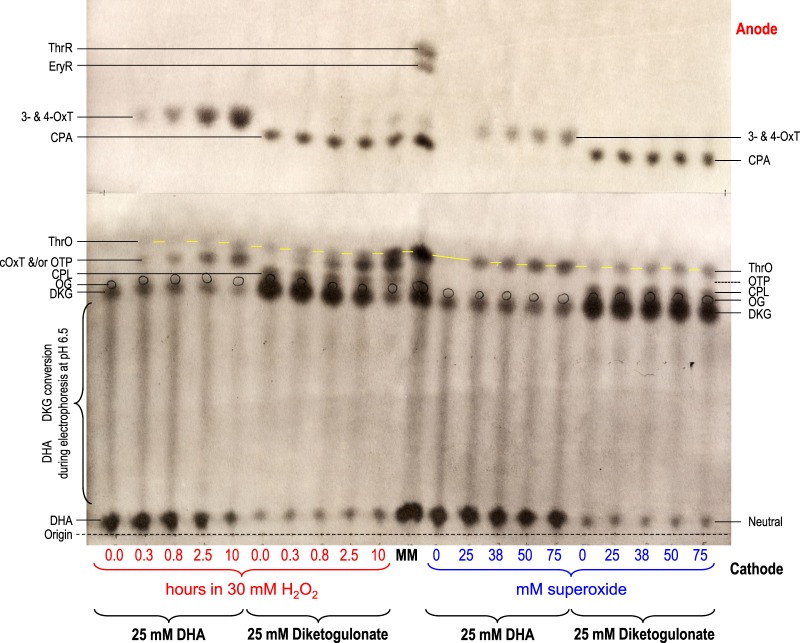
Reaction of dehydroascorbic acid and diketogulonate with H_2_O_2_ and superoxide: electrophoresis at pH 6.5. All details as for Figure 4a, but electrophoresis was conducted at pH 6.5 and 2.5 kV for 42 min.

DHA was incubated with a small excess of H_2_O_2_ (buffered at a typical apoplastic pH, 4.7), and products were analysed by electrophoresis at pH 2.0 (Figure 4) and 6.5 (Figure 5). At pH 6.5, all –COOH groups are almost fully charged (–COO^−^), causing migration towards the anode. In contrast, at pH 2.0, only those with an unusually low p*K*_a_ (e.g. OxA, cOxT, OxT, DKG, CPL, CPA and OTP) are highly mobile; however, many of the DHA and DKG products found in the present work fell in this category, so electrophoresis at pH 2.0 was valuable. ThrO is a weak acid, migrating very slowly at pH 2.0. DHA itself is immobile in both electrophoresis systems. All the singly ionised compounds of interest (DKG, cOxT, CPL, OTP and ThrO) formed a rather tight cluster on electrophoresis at pH 6.5, but were very well resolved at pH 2.0. The conclusions below are thus primarily drawn from Figure 4, but Figure 5 is compatible with them.

DHA in water gradually hydrolyses to DKG, more rapidly at higher pH values [82]. At pH 4.7 (our reaction mixture), hydrolysis is slow (half-life ≈ 8 h; [82]), but a small proportion of DKG was formed in ROS-free DHA samples (Figure 4). On electrophoresis at pH 6.5 (Figure 5), ROS-free DHA produced a streak between the DHA and DKG positions, due to partial hydrolysis of DHA during the 42-min run at that pH (half-life of DHA ≈ 20 min; [82]). There was no such streak on the pH 2.0 electrophoretogram, indicating negligible DHA hydrolysis.

The major stainable product formed during incubation of DHA with H_2_O_2_ was OxT (Figure 4), a reaction with the stoichiometry:

$${\text{C}}_{6}{\text{H}}_{6}{\text{O}}_{6}+{\text{H}}_{2}{\text{O}}_{2}\to {\text{C}}_{6}{\text{H}}_{8}{\text{O}}_{8}.$$

Electrophoresis at pH 2.0 (Figure 4) resolved at least two mutually interconvertible isomers: 3-OxT and 4-OxT [17,18,78]. Our reaction mixture initially contained 1.2 mol H_2_O_2_ per mol DHA, which is theoretically sufficient to give 100% oxidation of DHA to OxT, and indeed, H_2_O_2_ did gradually give a very high yield of OxT (Figure 4).

Only a faint trace of OxA was formed from DHA (Figure 4b), as revealed by staining with a pH indicator (for comparison, the same electrophoretogram, subsequently stained with AgNO_3_, is shown in Supplementary Figure S4. An unidentified spot (‘unk 2’; *m*_OG_ 0.53) was a contaminant of the commercial DHA and was seen equally in all samples, including in the DKG which we prepared from the DHA (Figure 4b).

Minor products of H_2_O_2_ action on DHA were cOxT and traces of ThrO, the latter detectable only after 10 h incubation. These have the same oxidation state as OxT (each ThrO assumed to be accompanied by an OxA molecule). The ratio of cOxT : OxT :  (ThrO + OxA) generated will depend on which bond(s) in the initial DHA oxidation product (proposed to be the short-lived cyclic 2,3-*O*-oxalyl-l-threonolactone; [17]) undergo hydrolysis (Figure 3). OxT and cOxT were formed concurrently, supporting the conclusion [17] that these two substances are formed from DHA independently (cOxT ← DHA → OxT), not sequentially (DHA → cOxT → OxT).

A minor product when DHA was treated with H_2_O_2_ was OTP, generated by the action of H_2_O_2_ on the DKG that was gradually formed by DHA hydrolysis (DHA → DKG → OTP). Some of the observed trace of ThrO may have arisen by further oxidation of this OTP (see below).

#### Superoxide gives the same DHA products as H_2_O_2_ but in a different ratio

DHA was also partially oxidised by ‘superoxide’ [at the pH of our reaction buffer, a ∼50 : 50 mixture of HO_2_^·^ and ${\text{O}}_{2}^{\cdot -}$, simplified in equations as $\text{H}{\text{O}}_{2}^{\cdot }$] (Figure 4). One mol of ‘superoxide’ can theoretically oxidise up to 1.5 mol of DHA to OxT:

$$3{\text{C}}_{6}{\text{H}}_{6}{\text{O}}_{6}+2{\text{HO}}_{2}^{\cdot }+2{\text{H}}_{2}\text{O}\to 3{\text{C}}_{6}{\text{H}}_{8}{\text{O}}_{8}.$$

However, this theoretical stoichiometry would be impossible for two reasons: first, superoxide has a very short half-life, undergoing dismutation

$$2{\text{HO}}_{2}^{\cdot }\to {\text{H}}_{2}{\text{O}}_{2}+{\text{O}}_{2}$$

(in competition with DHA oxidation), and secondly, the H_2_O_2_ by-product would have been rapidly removed by the added catalase.

Thus, it is impossible to quote a specific time-period for the reaction of the DHA with superoxide. The half-life of 50 mM superoxide at pH 4.7 is <1 µs (rate constant for dismutation at that pH, *k* ≈ 3 × 10^7^ M^–1^ s^–1^) [34,83]. Those DHA molecules that failed to react with superoxide within a millisecond of superoxide addition would thus have been spared any further oxidation by superoxide. Since time-course experiments were impossible, we instead carried out a dose–response study with 0–75 mM superoxide, each concentration being tested for the minimum possible duration, in the presence of catalase, and then froze the products.

Oxidation of DHA by superoxide, even when three molar equivalents (mol eq) of this ROS were added, was less complete than with 1.2 mol eq H_2_O_2_, and only a small proportion of the DHA disappeared (Figure 4). Superoxide, like H_2_O_2_, gave OxT (both isomers) as the major oxidation products plus a small amount of ThrO + OxA (Figure 4a,b). H_2_O_2_ gave the higher OxT yield, whereas superoxide gave the higher ThrO + OxA yield. However, unlike with H_2_O_2_, superoxide gave no detectable cOxT or OTP (Figure 4). The lack of OTP production supports the idea that the OTP formed by H_2_O_2_ (see previous section) had arisen from the small amount of DKG that was formed as a contaminant in the DHA solution (since superoxide does not generate OTP from authentic DKG; see section below). Therefore, the ThrO formed by superoxide must have arisen by an oxidation-then-hydrolysis route from DHA (e.g. DHA → cOxTL → ThrO + OxA; [17]) and not via the hydrolysis-then-oxidation route (DHA → DKG → OTP → ThrO; Figure 2e) mentioned above.

Thus, in general, superoxide generated smaller quantities of, and fewer different, DHA oxidation products than did H_2_O_2_.

#### Products of H_2_O_2_ action on DKG

DKG is formed from apoplastic AA, via intermediary DHA [14], and so has the potential to participate in reactions with apoplastic ROS.

Samples of DKG, prepared by brief alkali treatment of DHA and not subjected to ROS treatment, were contaminated by small amounts of OxA, CPA, CPL, unknown 1, ThrO, and neutral material (probably lactones of some of the above) (ROS-free samples in Figures 4 and 5). Most of these by-products did not decrease during treatment with H_2_O_2_ and superoxide, so they were not themselves ROS scavengers. Likewise *in vivo*, CPA and CPL are not oxidised (Figure 7 of ref. [17]).

DKG was gradually degraded by 1.2 mol eq H_2_O_2_, such that after 2.5 h roughly half remained (Figure 4). This was a similar % h^–1^ rate to the reaction of DHA with H_2_O_2_.

The major product formed from DKG by H_2_O_2_ was OTP. Once a significant pool of OTP had accumulated, ThrO also gradually appeared, compatible with the pathway DKG → OTP → ThrO. Both these steps are oxidative decarboxylations (Figure 2e), requiring an additional oxygen atom, ‘[O]’, provided by the H_2_O_2_:

$$\text{DKG}\;{\text{C}}_{6}{\text{H}}_{8}{\text{O}}_{7}+[\text{O}]\to {\text{C}}_{5}{\text{H}}_{8}{\text{O}}_{6}+\text{C}{\text{O}}_{2};$$

$$\text{OTP}\;{\text{C}}_{5}{\text{H}}_{8}{\text{O}}_{6}+[\text{O}]\to {\text{C}}_{4}{\text{H}}_{8}{\text{O}}_{5}+\text{C}{\text{O}}_{2}.$$

Since we supplied only 1.2 mol of H_2_O_2_ per mol of DKG, it would have been impossible for both the above reactions to go to completion, which would have required at least 2.0 mol/mol. When higher doses of H_2_O_2_ were supplied, a more complete conversion to ThrO was observed [18]. Thus, DKG has twice the capacity of DHA for scavenging H_2_O_2_.

An additional minor product was also formed from DKG, with electrophoretic mobilities close to those of 4-OxT (Figure 4). As expected, cold alkali hydrolysed it to ThrO (and probably OxA, detected with lower sensitivity; Supplementary Figure S3c). Interestingly, only one isomer was detected (4-OxT) in the DKG products, whereas DHA yielded both 3- and 4-OxT (Figure 4). This difference is clearest if the two samples with approximately the same 4-OxT yield are compared: the 10-h products of DKG + H_2_O_2_ *versus* the 0.3-h products of DHA + H_2_O_2_ (Figure 4). cOxT was not detected as a product of DKG.

#### Products of superoxide action on DKG

Superoxide did not perceptibly decrease the DKG concentration, even when a 3 : 1 superoxide : DKG ratio was tested (Figure 4). Nevertheless, traces of oxidation products were produced — the same range of products as with DKG + H_2_O_2_. These products included traces of 4-OxT, OTP, and ThrO. Thus, DKG is a very poor scavenger of superoxide.

### Reaction of ascorbate metabolites with Fenton reagent (source of hydroxyl radical)

#### Control experiments with Fenton mixture

We investigated the effects of ‘Fenton mixture’ (an equimolar mixture of FeSO_4_, EDTA, and H_2_O_2_, which generates ^•^OH) on DHA. The EDTA helps to keep the iron soluble (as Fe^2+^·EDTA), and the other components are expected to undergo a Fenton reaction:

$$\text{F}{\text{e}}^{2+}+{\text{H}}_{2}{\text{O}}_{2}\to \text{F}{\text{e}}^{3+}{+}^{∙}\text{OH}+\text{O}{\text{H}}^{-}.$$

The electrophoretic behaviour of Fenton mixture and its components, in the absence of DHA, is shown in Supplementary Figure S5. In control experiments with no added H_2_O_2_ but under aerobic conditions, 1–50 mM Fe^2+^·EDTA caused negligible oxidation of 50 mM DHA, as indicated by the absence of OxT and cOxT among the products (Supplementary Figure S6). Unexpectedly, high concentrations of Fe^2+^·EDTA (12.5–50 mM) promoted DHA → DKG hydrolysis (Supplementary Figure S6).

#### Reaction of DHA with complete Fenton mixture

**Figure 6.**
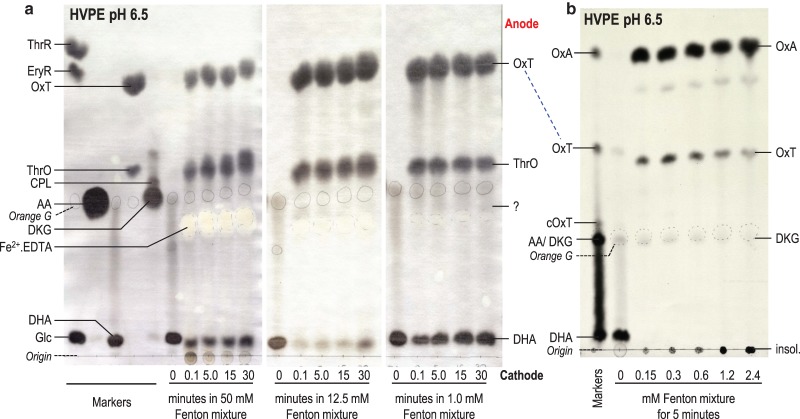
Reaction products of DHA with Fenton mixture. (**a**) DHA (50 mM) was incubated with a source of ^•^OH [Fenton mixture: 50, 12.5, or 1 mM each of EDTA, FeSO_4_, and H_2_O_2_ in 0.1 M acetate (Na^+^, pH 4.8)]. After 0–30 min, any further ^•^OH action was stopped by the addition of ethanol (to 50%). The samples (10 µl) were electrophoresed at pH 6.5 and products stained with AgNO_3_. (**b**) Approximately 0.3 mM [^14^C]DHA was incubated with increasing doses of Fenton mixture [equimolar FeSO_4_, EDTA, and H_2_O_2_; concentrations as indicated], for 5 min. Any further ^•^OH action was then stopped by the addition of EtOH (to 50%) and samples were electrophoresed at pH 6.5 and autoradiographed. The electrophoresis run-time was curtailed, so that OxA would remain on the sheet. The marker was an artificial mixture of ^14^C-labelled AA products. The position of the internal marker orange G is marked in pencil.

Fenton mixture products are summarised in Table 1. Remarkably, even the lowest tested concentration of the Fenton mixture (1 mM Fe^2+^·EDTA, 1 mM H_2_O_2_) was able to destroy the majority of a 50-fold molar excess of DHA within ‘6 s’ (Figure 6a), indicating that the Fenton mixture produced unlimited amounts of ROS, presumably ultimately from atmospheric O_2_. Higher concentrations of Fenton mixture (12.5 and 50 mM) acting on 50 mM DHA produced the same products as with the 1 mM mixture.

The reaction of DHA with Fenton mixture was not demonstrably time-dependent (0.1 and 30 min gave the same product yield; Figure 6a), although the DHA was never completely consumed. This suggests that the ethanol, added to scavenge ^•^OH after a chosen time-point, failed to prevent the reaction of earlier-formed (or continuously formed) oxidant(s) with DHA; thus, each sample effectively received the same (prolonged) oxidation time. The ethanol used (final concentration ∼10 M) was a 400-fold molar excess over the DHA and would thus have scavenged essentially all ^•^OH produced after the moment of ethanol addition (given that DHA and ethanol have comparable rate constants for reaction with ^•^OH [84]). However, the ethanol evidently did not stop the production or action of the highly effective, unidentified oxidant(s) that were generated by Fe^2+^·EDTA + H_2_O_2_ in the presence of O_2_.

Stainable products formed within ‘0.1 min’ from the reaction of 50 mM non-radiolabelled DHA with 1 mM (0.02 mol eq) Fenton mixture were predominantly OxT (Figure 6a), as in the case of DHA's reaction with H_2_O_2_ or superoxide. However, we know from Figure 4 that neither H_2_O_2_ (1.2 mol eq, e.g. for 48 min) nor superoxide (up to 3 mol eq) was capable of giving such a high yield of OxT. Therefore, the oxidant(s) responsible for DHA oxidation in the presence of 0.02 mol eq of Fenton mixture are unlikely to have been H_2_O_2_ or superoxide; they remain unidentified.

Besides OxT, a spot co-migrating with ThrO was also produced (Figure 6a). Use of [1-^14^C]DHA as a substrate confirmed that this spot was not the approximately co-migrating cOxT, which would have been radioactive [14] (Figure 6b). Unlike cOxT, ThrO (formed in the pathway [1-^14^C]DHA + [O] → ThrO + [^14^C]OxA) is not expected to be radiolabelled, because it does not retain the C-1 atom of the DHA [14]. Indeed, by far the most prominent radioactive product formed from [^14^C]DHA by Fenton mixture was [^14^C]OxA. The predominant reaction products formed from DHA by dilute Fenton mixture were thus OxA and ThrO, far exceeding the formation of the same products by H_2_O_2_ or superoxide.

We conclude that the Fenton mixture does not generate only ^•^OH. Other oxidant(s) were evidently also formed, ultimately drawing on atmospheric O_2_. These oxidising species oxidised DHA predominantly to OxA plus ThrO (Figure 6), unlike H_2_O_2_ or superoxide which gave OxT and cOxT (Figure 4a,b).

#### Reaction of DKG with Fenton mixture

**Figure 7.**
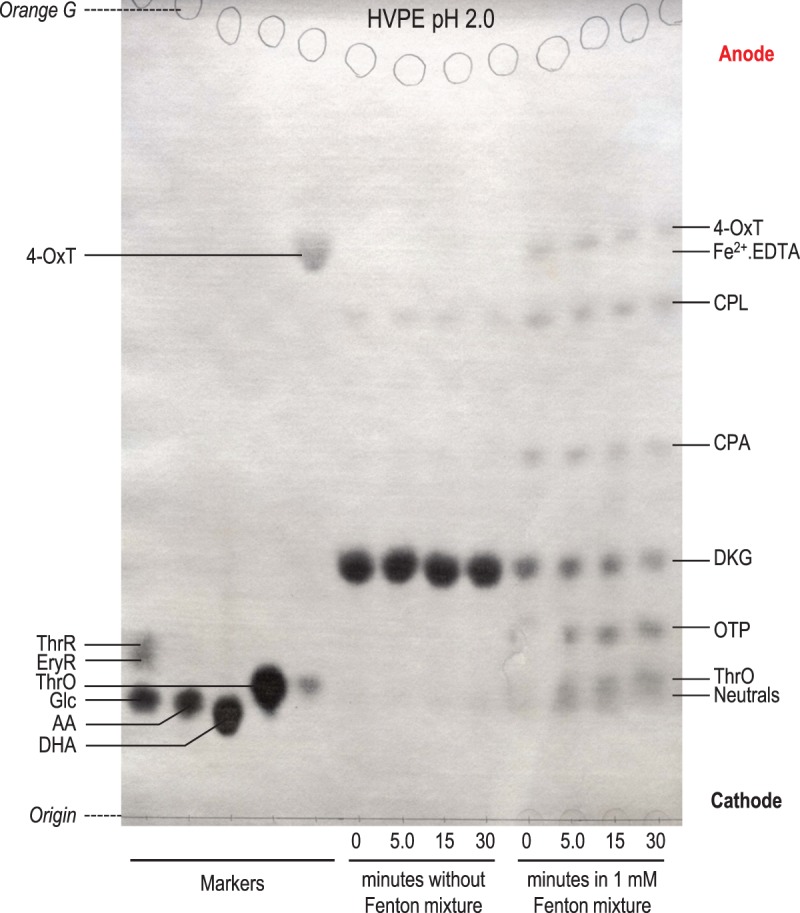
Reaction of DKG with Fenton mixture. Non-radioactive DKG [50 mM in 0.1 M acetate (Na^+^, pH 4.8)] was incubated with Fenton reagent (1 mM each of EDTA, FeSO_4_, and H_2_O_2_) for up to 30 min, after which further ^•^OH action was stopped by the addition of ethanol (to 50%). This DKG was prepared by the potassium iodate method. The time-0 sample represents DKG with ethanol added instantly after the Fenton reagents. Samples were electrophoresed at pH 2.0 and the products stained in AgNO_3_. The position of internal marker orange G is circled in pencil.

The reaction of 1 mM Fenton mixture with 50 mM DKG was also studied (Figure 7). In the absence of ROS, DKG was essentially stable for 30 min (Figure 7). As with DHA (Figure 6), a large proportion of the DKG was consumed by 0.02 mol eq of Fenton mixture, indicating an ‘inexhaustible’ source of oxidant (ultimately atmospheric O_2_). As with DHA, the Fenton mixture appeared to act ‘instantaneously’, degrading all the (50-fold molar excess) DKG even if ^•^OH-quenching ethanol was added immediately after the Fenton mixture.

The major products formed from DKG by Fenton mixture were ThrO, 4-OxT, and OTP. In addition, CPA and CPL (non-oxidative rearrangement products of DKG) were produced. There was no major difference between the observed Fenton products (Figure 7) and those produced from DKG by H_2_O_2_ or superoxide (Figure 4).

### The reaction of DHA and DKG with singlet oxygen, ^1^O_2_

**Figure 8.**
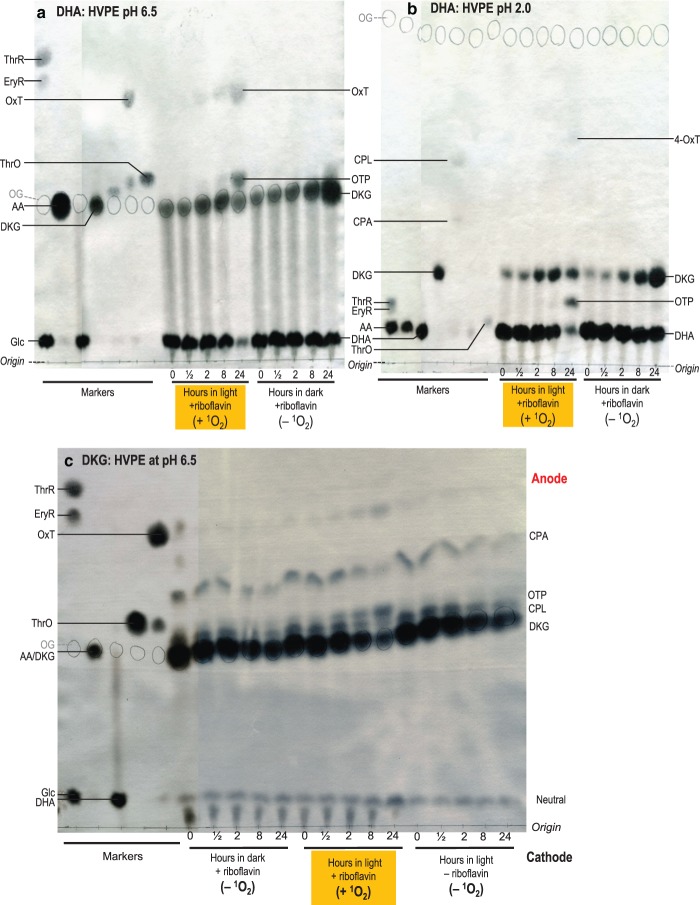
Reaction of AA catabolites with singlet oxygen. DHA at 50 mM in 0.1 M acetate (**a**,**b**) or DKG at 25 mM in 0.16 M acetate (**c**) (both buffers Na^+^, pH 4.8) was incubated with 1 mM riboflavin in the light (which generates ^1^O_2_) or in darkness. Samples taken at 0–24 h were electrophoresed at pH 6.5 (**a**,**c**) or 2.0 (**b**). Orange G (internal marker) was circled in pencil, then the metabolites were stained with AgNO_3_.

^1^O_2_ is produced by riboflavin (in the presence of air containing ordinary oxygen, ^3^O_2_) in the light but not in the dark [40,70,85], so samples incubated with riboflavin in the dark or without riboflavin in the light serve as ROS-free controls. ^1^O_2_ production from ^3^O_2_ by riboflavin in the light is not stoichiometric: each riboflavin molecule can generate many ^1^O_2_ molecules, although the yield is not unlimited because riboflavin itself is subject to oxidation by ^1^O_2_ [70].

We used riboflavin in the light to produce ^1^O_2_ over a longer timescale (24 h), allowing time for detectable ROS formation. This timescale allowed some of the DHA to hydrolyse to DKG during the experiment (Figure 8a,b); therefore, ‘DHA’ products could arise from DHA and/or DKG.

DHA plus ^1^O_2_ generated OTP and 4-OxT (Figure 8a,b). There was no detectable cOxT, CPA, or CPL. The formation of OxT and OTP, most prominent at 24 h incubation, coincided with the decrease in DKG, which was transiently formed by hydrolysis of the DHA during the lengthy incubation. Therefore, and since DKG tends to generate OTP when oxidised, we suggest that the products observed when DHA was treated with ^1^O_2_ were mainly derived indirectly, via DKG, rather than directly from DHA. A riboflavin-only control (Supplementary Figure S7) demonstrated that OTP and OxT did not originate from riboflavin itself.

Interestingly, there was no evidence for ThrO formation (the downstream product of OTP oxidation). Thus, ^1^O_2_ is incapable of driving an oxidative reaction that can be driven by H_2_O_2_ and to a lesser extent by superoxide (Figure 3).

When a sample of DKG (containing traces of CPA and CPL, which did not change in response to ^1^O_2_) was treated directly with the source of ^1^O_2_, most of the DKG disappeared within 24 h (Figure 8c). OTP and OxT were indeed formed, as detected by electrophoresis at pH 6.5 (Figure 8c), supporting the conclusion that the products formed from DHA (Figure 8a,b) predominantly arose via DKG.

## Discussion

### Further elucidation of the oxidation products of DKG

Ascorbate is the major low-molecular-weight, water-soluble, biological antioxidant. The fate of AA when acting as an antioxidant is well characterised [12,86]. AA is oxidised to monodehydroascorbate or DHA, which can be recycled to AA via MDHA reductase and DHA reductase, respectively. Alternatively (perhaps especially in the apoplast), DHA can be hydrolysed to DKG; both DHA and DKG have been detected *in vivo* [14]. In the apoplast, in the absence of DHA reductase recycling DHA back to AA, DHA and DKG are themselves also capable of scavenging ROS, as confirmed in the current work. However, the products of oxidation of DHA and DKG had not been fully elucidated, especially those formed in the presence of ROS other than H_2_O_2_. We now report two new conclusions about the oxidation products of DKG.

First, DKG can give low yields of 4-OxT when treated with ROS; this finding refines the previous assumption that OxT arises from DHA oxidation and not from DKG. The new step (Figure 1, dashed line) gives 4-OxT unaccompanied by 3-OxT or cOxT, and may therefore not proceed via [cOxTL], the short-lived intermediate proposed as a branch-point during DHA oxidation. To account for the specific formation of 4-isomer, we speculate that the ROS reacts with DKG in its minor ε-lactone form, oxidatively splitting the C-2–C-3 bond and leaving the resultant oxalyl residue (former C-1 and C-2) esterified to the former C-6. Oxidatively splitting the C-2–C-3 bond in the better-known δ-lactones [22] would probably afford 3-OxT (i.e. with the oxalyl residue esterified to the former C-5).

Secondly, we identified the DKG product previously called ‘unknown **H**’ [18] as 2-oxo-l-*threo*-pentonate (OTP; ‘2-keto-l-xylonate’), the oxidative decarboxylation product of DKG. OTP is a relatively strong acid (p*K*_a_ ≈ 2.8), as expected of a 2-oxo-acid, and thus occupies a characteristic position after electrophoresis at pH 2.0.

The work thus provides increased clarity of the complex network of AA oxidation pathways, highlighting the numerous different branches.

### Testing effects of superoxide with minimal interference by H_2_O_2_

Previous work on oxidation of AA metabolites had focussed on H_2_O_2_. Here, we tested the effects of different ROS. Studying superoxide's action presents two practical difficulties: (a) superoxide is exceedingly short-lived in aqueous solution (half-life <<1 s), undergoing non-enzymic dismutation, so time-courses are not feasible and (b) the products of superoxide breakdown are a different ROS (H_2_O_2_) plus O_2_, so it may be difficult to determine whether any products observed arise from the action of superoxide (intended) or H_2_O_2_ (unintended).

Crystalline KO_2_ is a convenient source of superoxide. KO_2_ was suspended in hexane and then added to an aqueous substrate solution. When mixed with water, KO_2_ fizzes briefly (O_2_ evolution during dismutation) and any superoxide action on DHA or DKG is necessarily completed within <1 s; it is therefore impossible to obtain a time-course. Instead, the only feasible gradation of superoxide action is based on different doses of added KO_2_, time being kept to a minimum. Another practical issue is that since KO_2_ + water yields KOH, we required a high buffer concentration.

To minimise the effects of H_2_O_2_, generated during superoxide dismutation, we added catalase prior to KO_2_ to destroy H_2_O_2_. Its effectiveness was established by a demonstration that H_2_O_2_ that was deliberately added to DHA or DKG in the presence of catalase generated no detectable oxidation products.

### Properties of the Fenton reagent

The classic Fenton reagent is an equimolar mixture of Fe^2+^ and H_2_O_2_, which generates ^•^OH [87,88]. Often, EDTA is also added, forming Fe^2+^·EDTA, which has improved solubility and which we find is stable enough to electrophorese as a discrete, negatively charged complex. Theoretically, a mixture containing 1 mol of each component may yield up to 1 mol of ^•^OH:

$$\text{F}{\text{e}}^{2+}+{\text{H}}_{2}{\text{O}}_{2}\to \text{F}{\text{e}}^{3+}{+}^{∙}\text{OH}+\text{O}{\text{H}}^{-}.$$

However, we find that 1 mM Fenton mixture very quickly (within 6 s) oxidises the majority of 50-fold excess DHA. It is therefore impossible to satisfactorily explore the effects of ^•^OH on DHA or DKG by use of Fenton reagent because this mixture evidently continually generates unidentified oxidant(s) using atmospheric O_2_ as the ultimate electron acceptor. The information recorded for DHA and DKG oxidation by Fenton mixture (Table 1) thus relates to an unknown oxidant, not specifically ^•^OH.

### Different ascorbate metabolites target specific ROS, producing different ratios of oxidation products: potential analytical fingerprints

Although there has been much interest in the ROS-scavenging potential of various antioxidants, especially AA but also DHA and occasionally DKG, few studies have sought to compare different AA metabolites' abilities to quench different ROS.

In contrast with intraprotoplasmic AA which can be recycled via the DHA reductase/glutathione pathway, further oxidation of DHA or DKG is sacrificial, resulting in the loss of antioxidant capacity. The oxidation of DHA or DKG is more likely to occur in the apoplast, or in cell components where reducing agents, such as glutathione, are less prevalent [89] and in which AA would therefore not be regenerated from DHA.

With **DHA**: H_2_O_2_ is scavenged effectively, generating 4-OxT, 3-OxT, and cOxT. Superoxide is scavenged slightly by DHA, giving 4-OxT and 3-OxT. However, ^1^O_2_ appears not to be scavenged by DHA at all (except insofar as DHA can be hydrolysed to DKG which then scavenges ^1^O_2_). With **DKG**: superoxide is scavenged only very weakly by DKG, which, however, scavenges ^1^O_2_ and H_2_O_2_ relatively well.

Similarly, we made the novel observation that the degradation pathways of DHA and DKG vary depending on the type of ROS present. This observation could be exploited to indicate the nature of oxidative stress that an organism is experiencing, based on the signature of the DHA and DKG oxidation products. Some studies in this field have used non-aqueous solvents, e.g. the action of superoxide on DHA to form oxalate + threonate in dimethylformamide [90], in which the reaction pathways may not be the same as *in vivo*. Our studies investigated only aqueous media.

For example, different ROS generate different OxT isomer ratios from DHA: H_2_O_2_ produces approximately equimolar 3-OxT and 4-OxT plus some cOxT but almost no OxA, whereas superoxide gives predominantly 4-OxT, only traces of 3-OxT and undetectable cOxT but some detectable OxA (with ThrO) (Figure 4). The reason for the H_2_O_2_ *versus* superoxide difference is unclear: since the divergence between cOxT, OxT, and OxA (and the conversion of 3-OxT to 4-OxT) is proposed to occur after the last ROS-dependent step (DHA → [cOxTL]; Figure 1), it is unclear how the nature of the ROS employed can direct this branching pathway, which involves hydrolysis and acyl-migration reactions. Nevertheless, the trend is clear cut.

In the case of DKG oxidation, H_2_O_2_ gives much more OTP than does superoxide, whereas these two ROS give similar (low) yields of 4-OxT (Figure 4). Thus again, there is a clear H_2_O_2_ *versus* superoxide difference.

Singlet oxygen, the only non-radical ROS tested in this study, shows the most divergent profile of oxidation products (Figure 8). Curiously, ^1^O_2_ seems incapable of oxidising DHA directly, though it does effectively oxidise DHA's gradually formed hydrolysis product, DKG. Acting directly on DKG, ^1^O_2_ (like H_2_O_2_) steadily yields OTP, whereas superoxide does not. Since DHA does not generate OTP, the yield of OTP can indicate whether or not a particular ROS is scavenged by DKG.

*In vivo* different ROS often occur together, and many ROS react to form other ROS, such as the production of ^1^O_2_ [74] and ^•^OH [73] from ozone, and ^•^OH from H_2_O_2_ and superoxide [34]. This can create uncertainty in investigated systems as to which ROS is contributing to the oxidative stress *in vivo*. Therefore, analysis of DHA and DKG oxidation ‘fingerprints’ may help to shed light on this important issue. The use of these diagnostic ‘fingerprints’ will require significant further study because of the complexity of ROS reactions in biological systems as well as the complexity of interactions with other molecules. *In planta* ROS-scavenging activity is likely to be influenced by other metabolites, and it would be useful to analyse the effectiveness of DHA and DKG as scavengers *in vivo* or *in planta*.

### Possible biological roles of specific DHA and DKG metabolites

It is possible that the various products formed from the reactions of different ROS with different AA metabolites could supply information, making the plant aware of its internal ROS status. Thus, the OxT isomers, cOxT and OTP, might potentially act as signalling molecules, triggering advantageous responses to the presence of specific ROS. Production of OTP from DKG + ^1^O_2_ may represent biologically relevant case of when OTP could be produced *in vivo*. Although H_2_O_2_ has been demonstrated to act as a signalling molecule for a general oxidative stress response, it has been suggested that plant cells may require more site-specific (e.g. from specific organelles) signals [91]. The different AA oxidation products from different ROS could provide this signal specificity; for instance, ^1^O_2_ derived products (different from H_2_O_2_ and superoxide products) are more likely to have originated from the chloroplast [41]. Equally, oxidation products of carotenoids have been recently demonstrated to influence expression of ^1^O_2_ related genes, but not H_2_O_2_ related genes [92]. The AA oxidation products described in this study could potentially play similar roles within the plant. With the exception of OxT [16], the oxidation products discussed have to date been detected *in vivo* mainly with the use of radiolabelled tracers, which should, nevertheless, reflect processes that occur with endogenous compounds.

Indeed, the formation of DKG itself, which has been detected *in vivo* [14,93], may also supply information about ROS status since high levels of DKG can only accumulate (from DHA hydrolysis) when DHA is not being rapidly oxidised by ROS. In plants, OxT and cOxT are also capable of transferring their oxalyl group to carbohydrates, catalysed by an apoplastic acyltransferase activity [20], and the roles of the resulting *O*-oxalyl-sugars are yet to be explored.

## Conclusion

Our study highlights the complexity of AA catabolism and demonstrates the numerous branches of AA, DHA, and DKG breakdown. The possibility of their reaction products having further roles within the plant warrants future study. The different AA metabolites investigated here differ in their abilities to scavenge different ROS. The oxidation products of DHA and DKG differ, with only DHA producing cOxT and 3-OxT, and only DKG producing OTP. Different ROS tend to generate different ratios of the oxidation products from these two AA metabolites. The diverse products could potentially act as biological signals enabling the organism to respond appropriately to different stresses. In addition, these products of DHA and DKG breakdown, which are readily resolved by the electrophoretic systems employed here, can potentially serve as analytical fingerprints providing information on the ROS-quenching reactions proceeding in biological systems of interest.

## Abbreviations

4-OxT: 4-*O*-oxalyl-l-threonate
AA: l-ascorbate
CPA: (formerly called compound **E**) proposed to be 2-carboxy-l-*threo*-pentonate (i.e. ‘2-carboxy-l-xylonate’ or ‘2-carboxy-l-lyxonate’, which are synonyms)
CPL: (formerly called compound **C**) proposed to be a mixture of 2-carboxy-l-*threo*-pentonolactones (2-carboxy-l-xylonolactone plus 2-carboxy-l-lyxonolactone)
cOxT: cyclic oxalyl-l-threonate
cOxTL: cyclic oxalyl-l-threonolactone
DHA: dehydro-l-ascorbic acid
DKG: diketo-l-gulonate
EDTA: ethylenediaminetetraacetate
EryR: erythrarate (*meso*-tartrate)
mol eq: molar equivalent(s)
OAH: 2-oxo-*arabino*-hexonate (2-keto-gluconate)
OTP: 2-oxo-l-*threo*-pentonate (‘2-keto-l-xylonate’)
OxA: oxalate
OxT: oxalyl-l-threonate
OxTL: oxalyl-l-threonolactone
ROS: reactive oxygen species
ThrO: l-threonate
ThrR: l-threarate (l-tartrate)
