# Supplementary material for: The oxidation of dehydroascorbic acid and 2,3-diketogulonate by distinct reactive oxygen species
Source: Biochem J. 2018 Nov 9;475(21):3451–70. doi: 10.1042/BCJ20180688 (PMC6225978; doi:10.1042/BCJ20180688)
Supplement: Supplementary Figures [file BCJ-475-3451-s1.pdf]

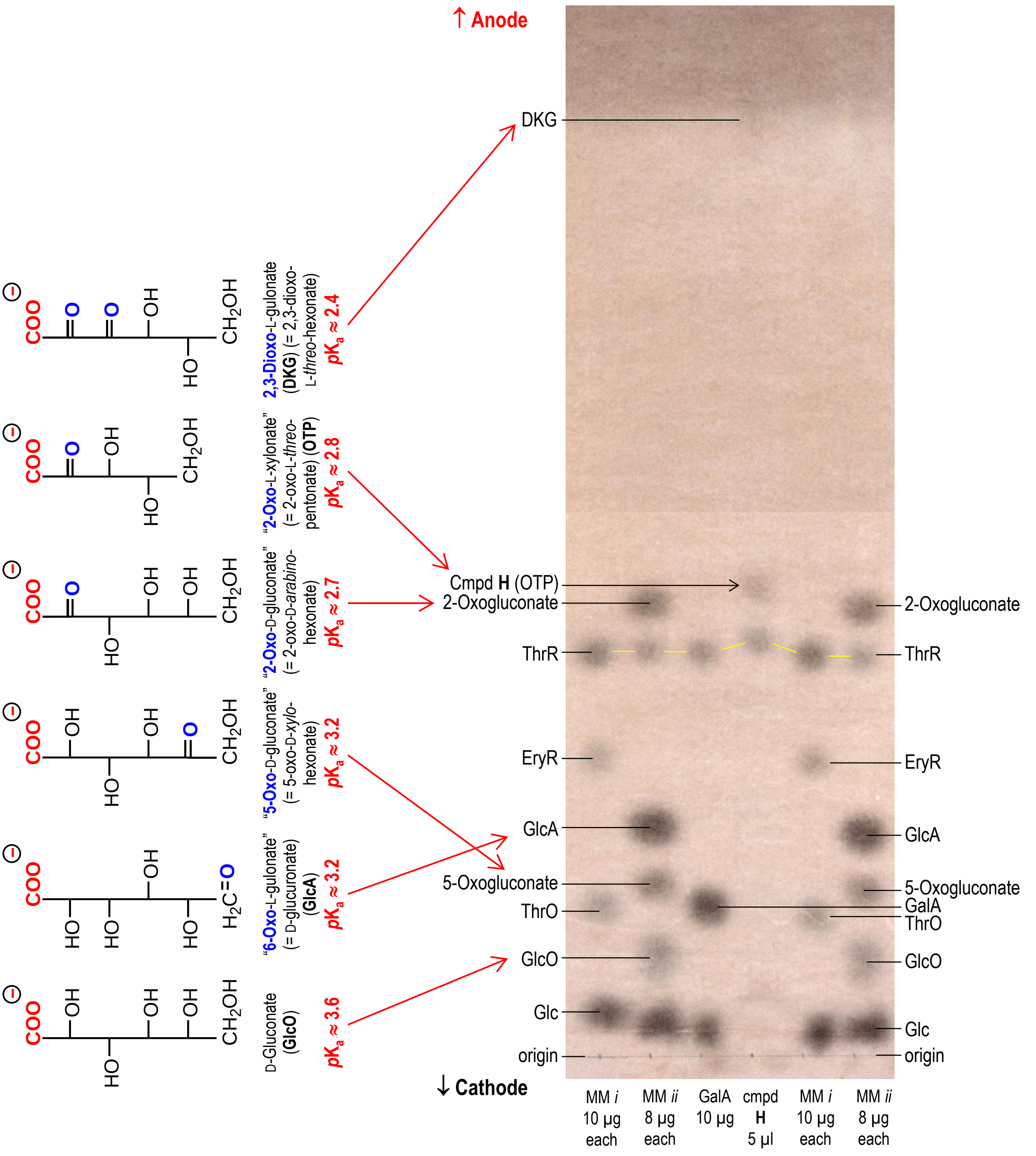

Fig. S1. Mobilities of standards and OTP (compound H) on high-voltage electrophoresis at pH 2.0.

The sample of compound **H** (in lane 4) was 5 µl of the 5-hour reaction products between DKG and H_2O_2 (for details, see legend to Fig. 4). MM *i* and *ii* = marker mixtures of authentic compounds: glucose (Glc), gluconic acid (GlcO), threonic acid (ThrO), galacturonic acid (GalA), 5-oxogluconate, glucuronic acid (GlcA), erythraric acid (EryR; *meso*-tartaric acid), threaric acid (ThrR; L-tartaric acid) and 2-oxogluconic acid. ThrR was added to all samples, including OTP (compound **H**), as an internal marker (see yellow lines).
Electrophoresis was at pH 2.0 and 3 kV for 3.5 h; the stain was silver nitrate. [The high sodium acetate buffer content of the compound **H** sample has distorted the position of neutral substances in this sample.]

Fig. S2

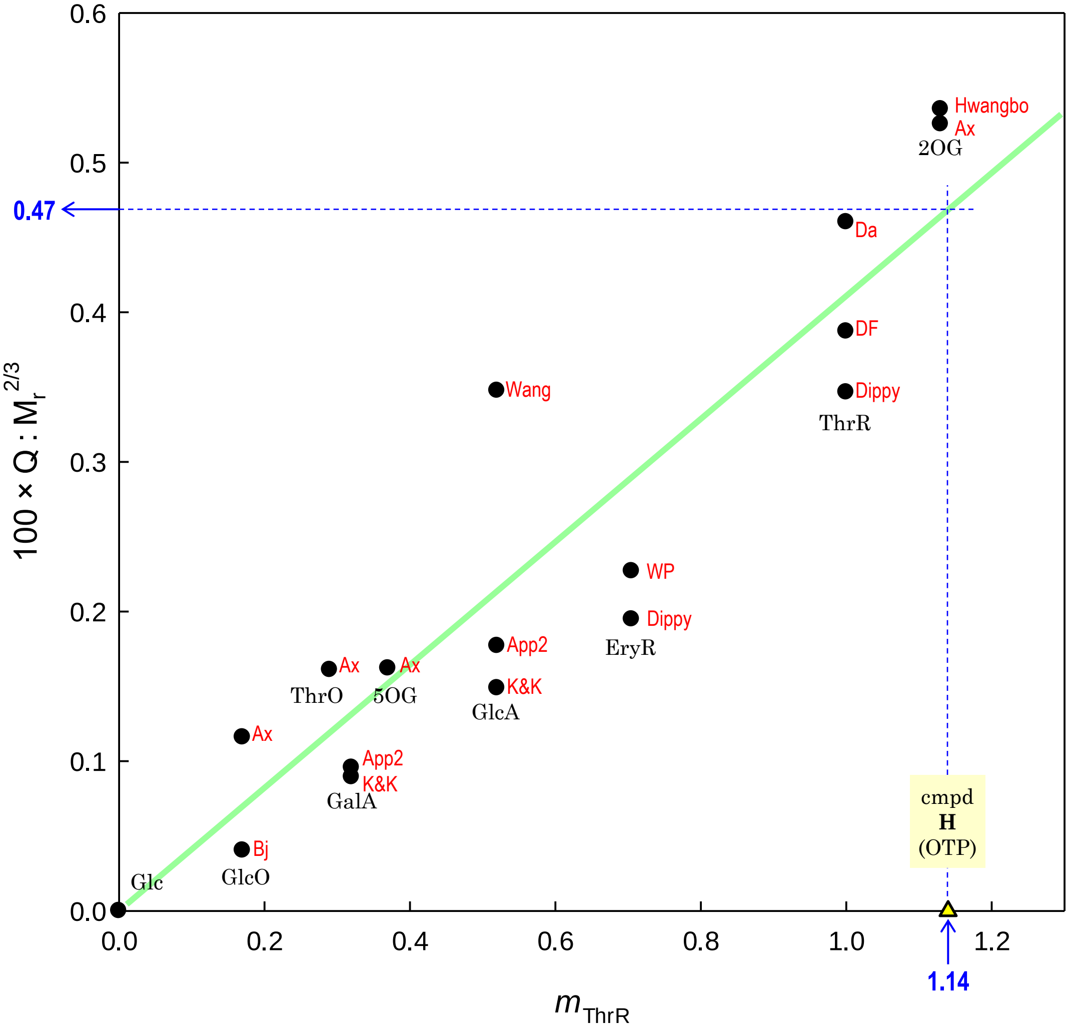

Black labels (compounds tested):
Glc, glucose
GlcO, gluconic acid
ThrO, threonic acid
GalA, galacturonic acid
5OG, 5-oxogluconic acid
GlcA, glucuronic acid
EryR, erythraric acid (*meso*-tartaric acid)
ThrR, threaric acid (L-tartaric acid)
2-OG, 2-oxogluconic acid
cmpd H, a diketogulonate product, shown here to be 2-oxo-*threo*-pentonate (OTP)

Red labels = source of published pK_a values:
WP: Wikipedia
Bj: Bjerrum et al. (1958)
Da: Dawson, R.M.C. et al., Data for Biochemical Research, Oxford, Clarendon Press, 1959.
App2: Appendix 2 of: Applications of Ion Chromatography for Pharmaceutical and Biological Products, First Edn. Ed by L.Bhattacharyya & J.S. Rohrer
K&K: Kohn & Kovác' (1978) Chem. Zvesti 32: 478–485 (1978
Ax: ChemAxon (prediction)
Wang: Wang et al., 1991, Biochem J 278: 689
Dippy: Dippy JFJ, Hughes SRC, Rozanski A (1959) J Chem Soc 1959, 2492.
DF: DrugFuture.com
Hwangbo: Hwangbo H., Park R.D., Kim Y.W., Rim Y.S., Park K.H., Kim T.H., Suh J.S., Kim K.Y., Curr. Microbiol., 47, 87–92 (2003).
*assumes same M_r = 164.

Fig. S2. Estimation of *p*K_a of OTP (compound H) by electrophoresis at pH 2.0.
Values of $Q : M_r^{2/3}$ [see note 1] at pH 2.0 for nine relevant compounds were calculated from published *p*K_a values and known molecular weights, then plotted against observed m_{ThrR} (the electrophoretic mobility at pH 2.0 relative to that of L-threarate). The green line is the linear regression of all data. The observed electrophoretic m_{ThrR} value of compound **H** (1.14; see Fig. S1) is indicated on the *x*-axis, and the estimated $100 \times Q : M_r^{2/3}$ was read off on the *y*-axis (0.47). This value indicates that the *p*K_a of compound **H** is 2.78 (if we assume that **H** is a 2-oxoaldonate; M_r = 164), which is close to the published *p*K_a values of 2-oxogluconate (≈ 2.7).

Notes
[1] The electrophoretic mobility of a small molecule is proportional to its $Q{:}M_r^{2/3}$ ratio (Offord, 1966; Fry, 2011) where *Q* is the net charge of the molecule at the pH of the electrophoresis buffer and molecular weight (M_r) to the power of $^2/_3$ is an indication of its relative surface area. Calculated $Q{:}M_r^{2/3}$ ratios [see note 2] of several relevant organic acids at pH 2.0, based on published *p*K_a values, are plotted against their observed electrophoretic mobility at that pH. Note that the published *p*K_a values for any given compound are rather inconsistent, but the trend line is clear, from which we estimate compound **H** to have a *p*K_a of about 2.8, which is close to the published value for 2-oxo-*arabino*-hexonate.
[2] For calculation of Q we derived the ratio of the anion (A^-) to the un-ionised acid (AH) from the equation:
$\log\{[A^-] / [HA]\} = pH - pK_a$

Fig. S3

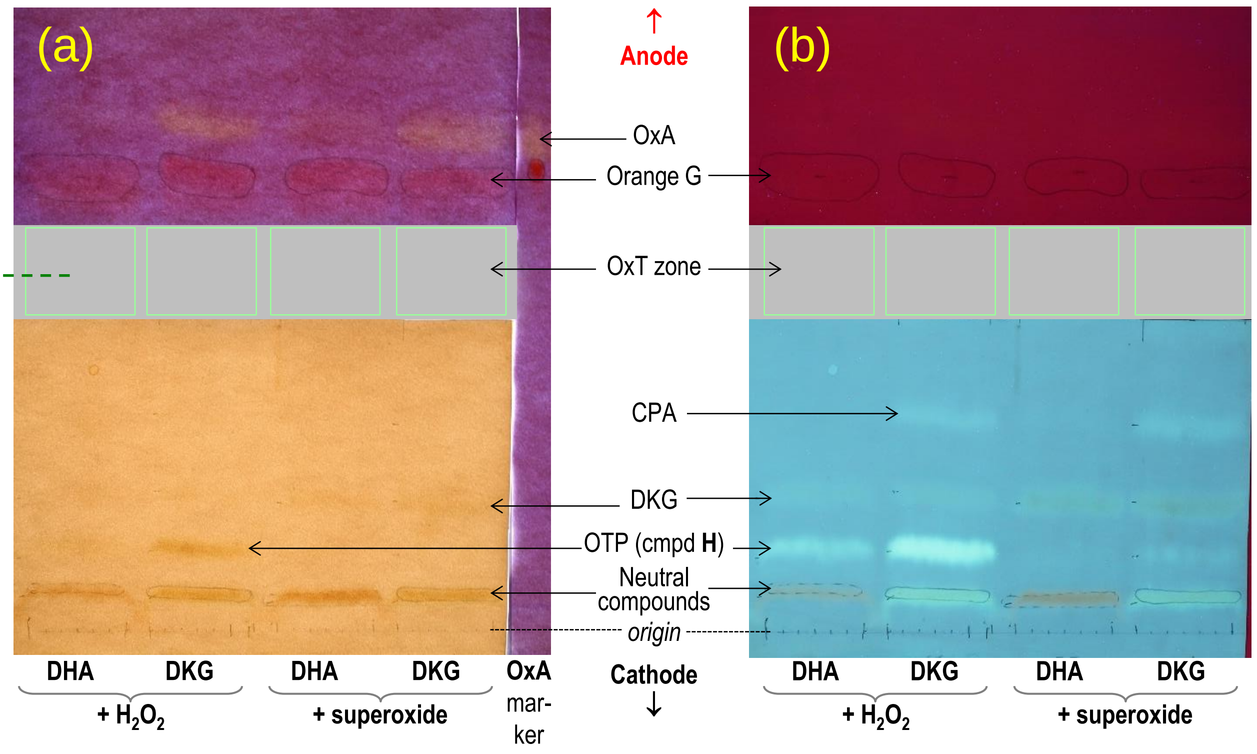

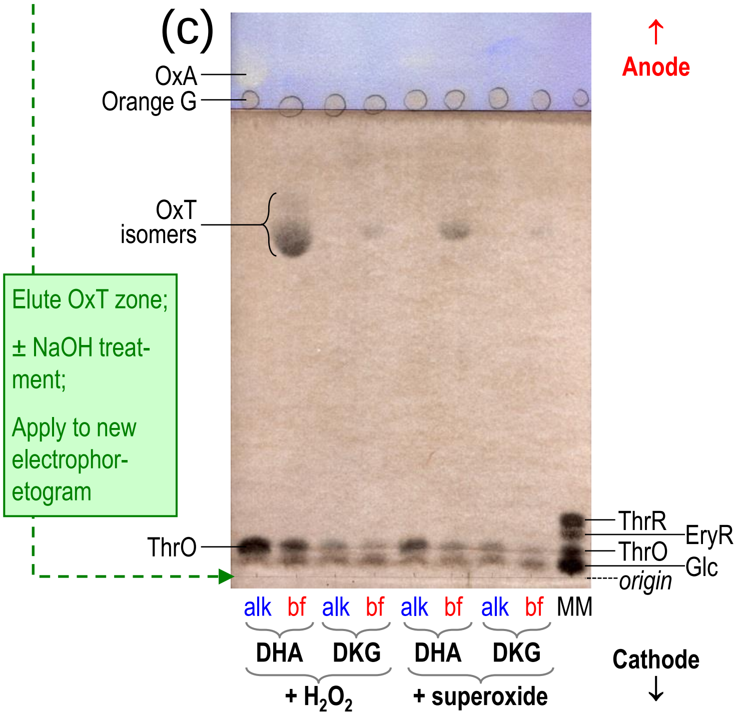

Fig. S3. Further characterisation of the products of ROS action on dehydroascorbic acid (DHA) and diketogulonate (DKG).

DHA and DKG were ROS-treated (10 h in 30 mM H_2O_2 or <1 min with 75 mM potassium superoxide), then the oxidation products were separated by semi-preparative paper electrophoresis (pH 2.0, 2.0 kV, 60 min). Sections of the paper were stained with aniline hydrogen-phthalate [yellow/brown background in (a)] or bromophenol blue [purple background in (a)], then viewed in white light (panel a) or under 360-nm UV (panel b). The OxT zone (marked in grey) was not stained; instead, the rectangles indicated in green were cut out and compounds present were eluted, treated with NaOH (alk) or buffer (bf), and re-run by electrophoresis as above (panel c).

It is seen (panel c) that all four ‘OxT’ zones were completely hydrolysed by alkali, yielding ThrO (stained with silver nitrate) plus OxA (faint yellow spots with bromophenol blue). [The buffer-treated controls had been partially hydrolysed to ThrO during the work-up, even without alkali treatment.] In addition, all the samples revealed a similar spot of neutral material (co-migrating with glucose) — probably contaminating carbohydrates eluted from the paper shown in (a) and (b).] MM, marker mixture. *Abbreviations:* CPA, 2-carboxy-L-*threo*-pentonate, formerly called compound E (Parsons *et al.*, 2011); DHA, dehydro-L-ascorbic acid; DKG, 2,3-diketo-L-gulonate; EryR, erythrarate (*meso*-tartrate); OTP, 2-oxo-*threo*-pentonate (compound **H**); OxT, 3- and/or 4-*O*-oxalyl L-threonate; ThrO, L-threonate; ThrR, L-threarate (L-tartrate).

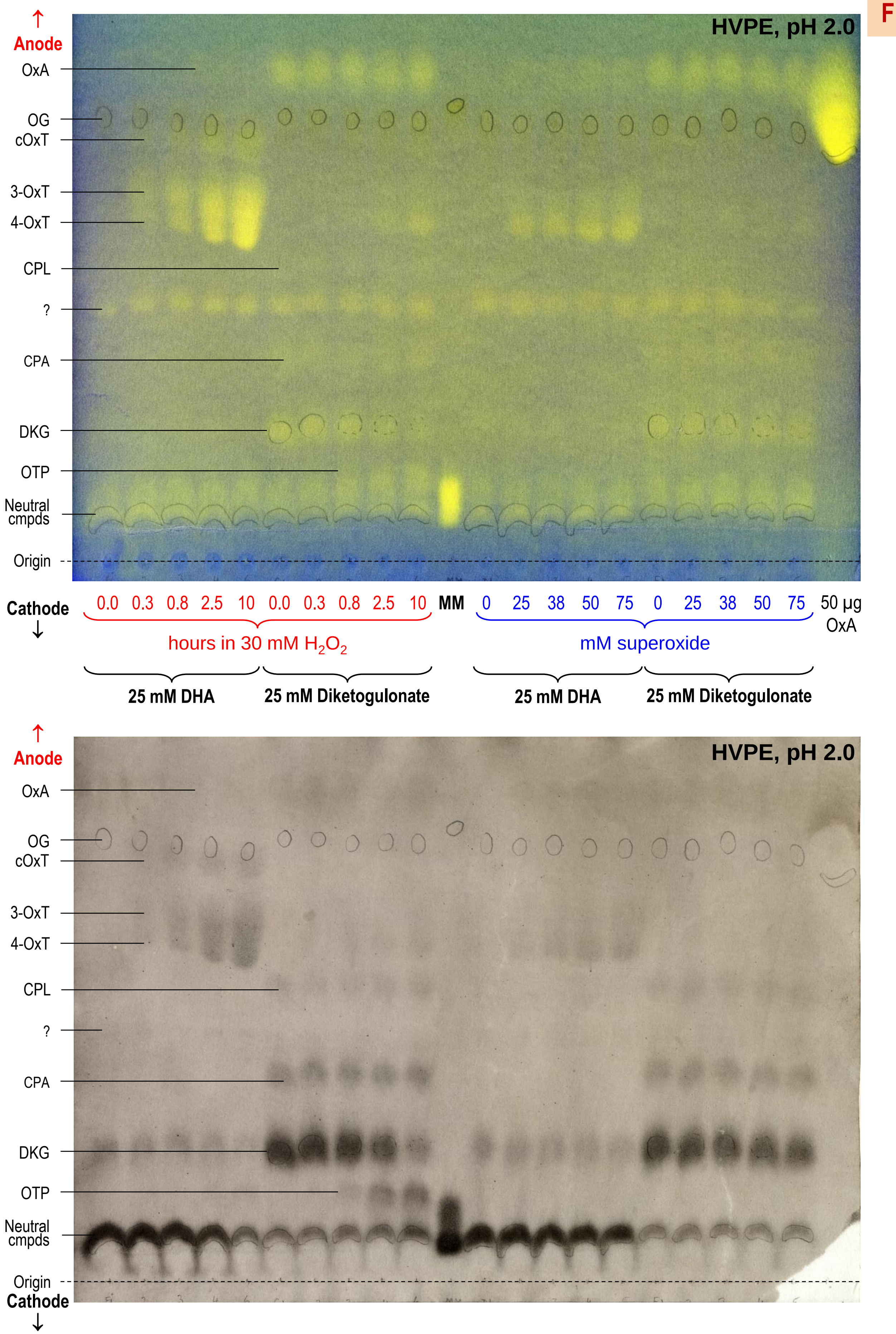

Fig. S4. Sequential staining of an electrophoretogram with bromophenol blue and silver nitrate. The bromophenol blue-stained electrophoretogram seen in Fig. 4b, showing total anionic products, is reproduced here (top), and after subsequent staining with $AgNO_3$ to reveal specifically the 'carbohydrate-related' compounds (bottom).

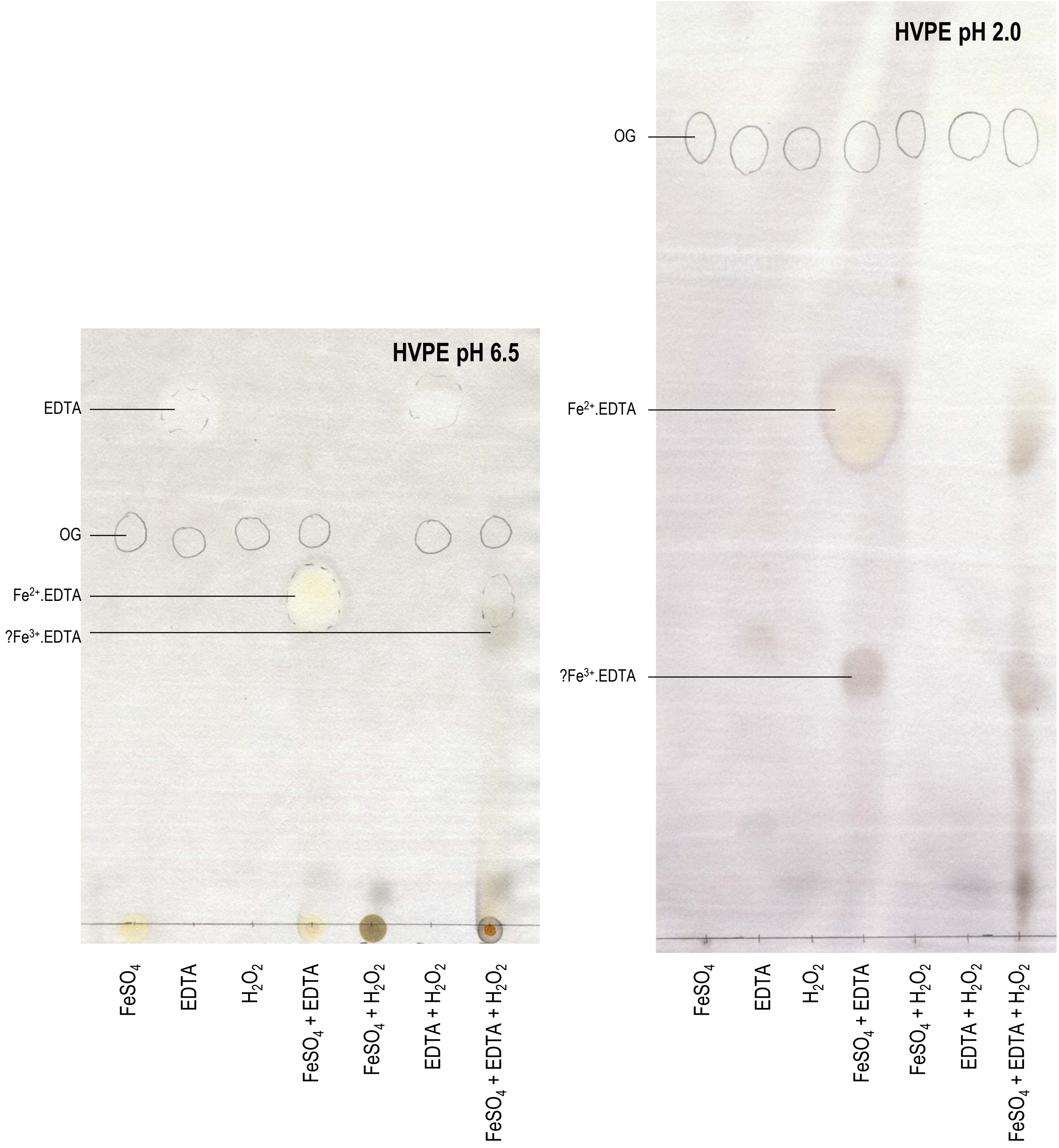

Fig. S5. Electrophoretic behaviour of $FeSO_4$, EDTA, H_2O_2, and combinations thereof.
Each compound indicated was present at 50 mM in 100 mM acetate (Na^+, pH 4.5), and 10 µl was applied to the electrophoretogram. electrophoresis was conducted at pH 6.5 (left) or 2.0 (right), and spots were stained with $AgNO_3$. Each sample contained a trace of Orange G (OG) as an internal marker, which was marked in pencil before silver-staining.

Fig. S6

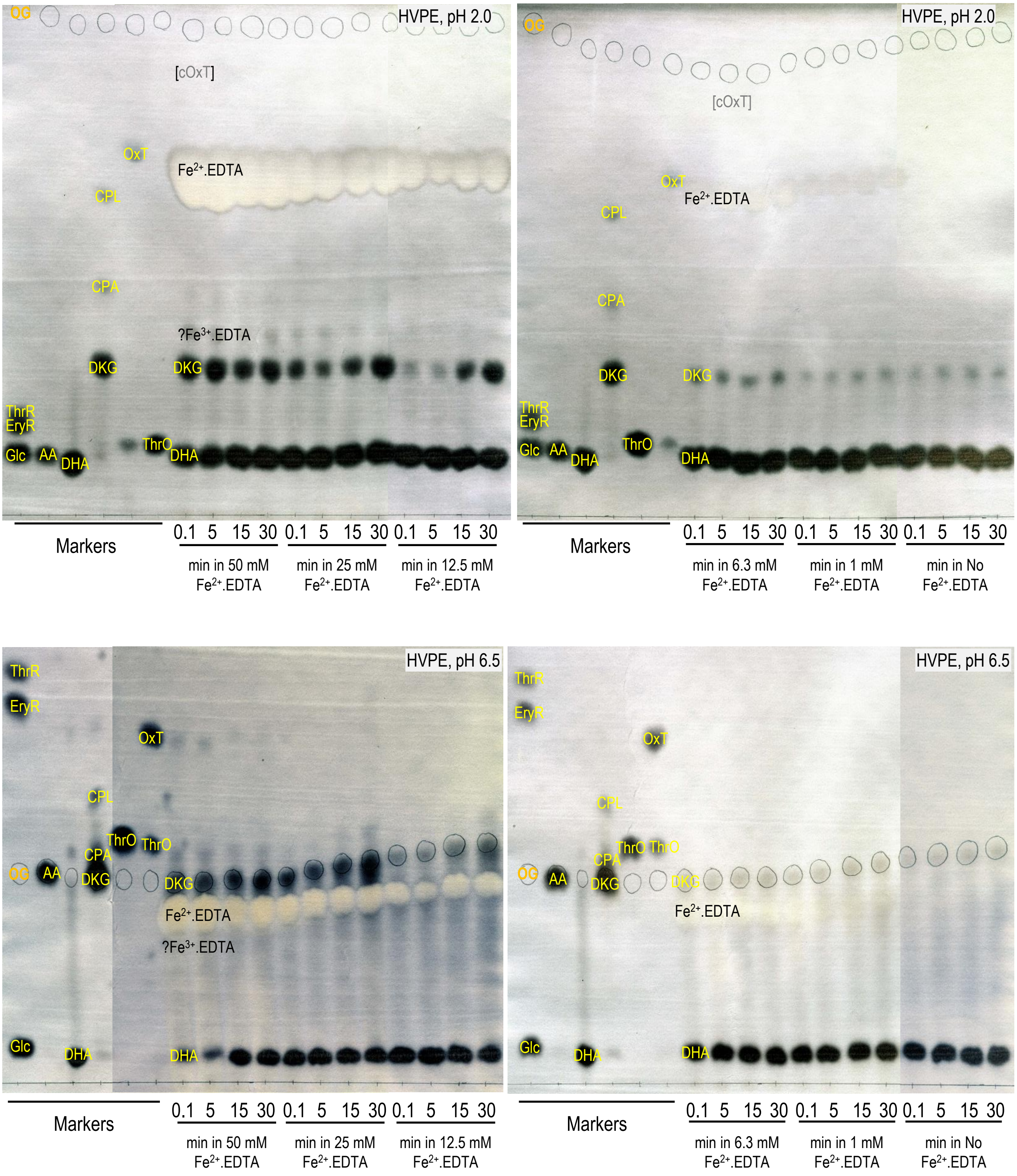

Fig. S6. Control experiment to test the effect of Fe^{2+}.EDTA on DHA in the absence of added H_2O_2

DHA (50 mM) was incubated for ~0.1–30 min with various concentrations of Fe^{2+}.EDTA in 100 mM acetate (Na^+, pH 4.5) in the absence of any deliberately added H_2O_2. At the desired time-points, a sample was added to ethanol (which quenches $^{\bullet}OH$) and then stored at –20°C. Portions of the products were then analysed by HVPE at pH 2.0 (above) or 6.5 (below). Markers and products were stained with $AgNO_3$; the Orange G (OG) internal marker was circled in pencil before staining. [cOxT] = position of cOxT but no spot visible. For abbreviations, see Fig. 4.

The electrophoretograms show that Fe^{2+}.EDTA in absence of H_2O_2 does not oxidise DHA (almost no OxT and no cOxT were formed). On the contrary, some DKG was formed, promoted by high Fe^{2+}.EDTA. DKG formation was time-dependent at moderate Fe^{2+}.EDTA concentrations (12.5, 25 mM).

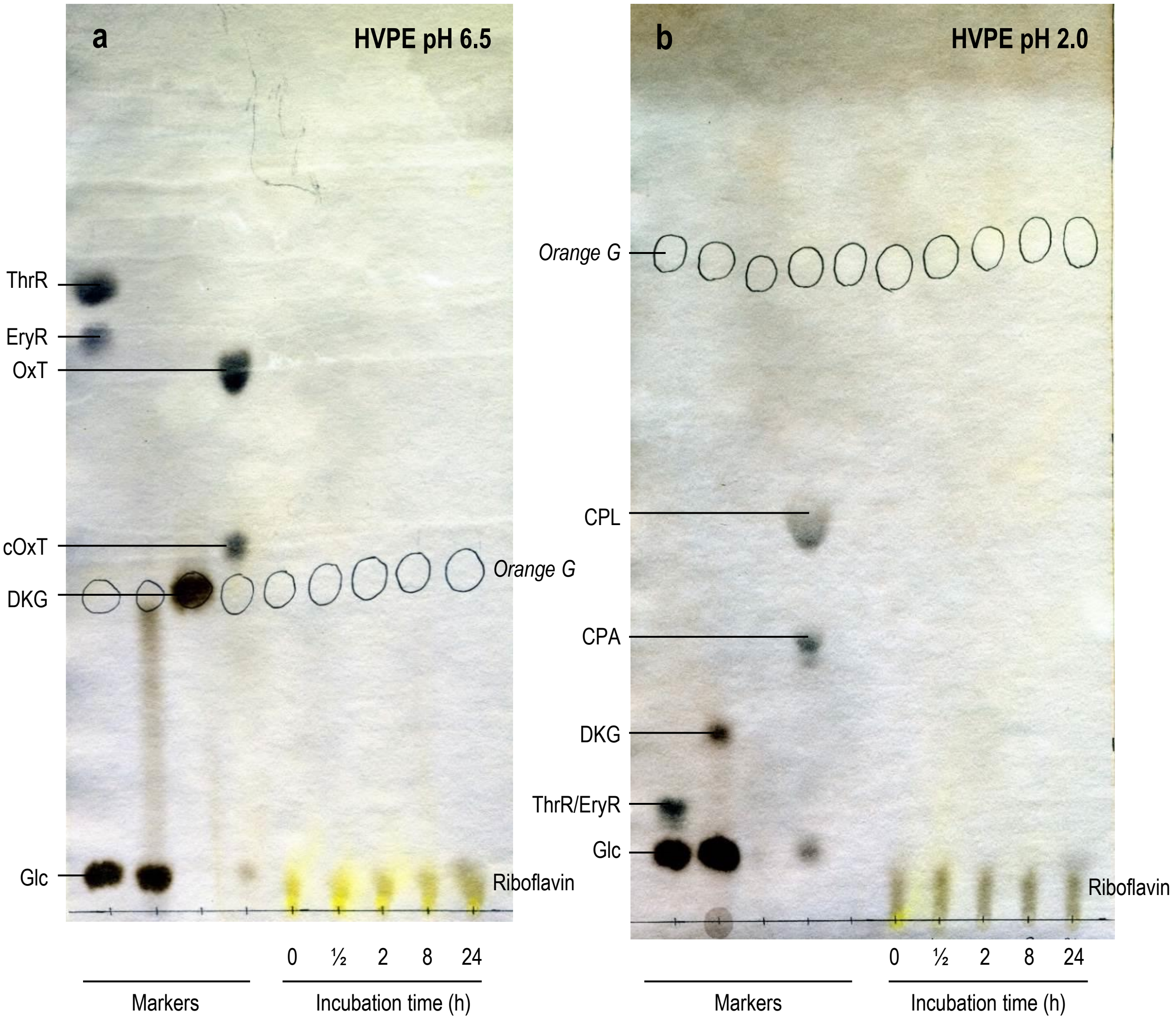

Fig. S7: Compounds formed during the degradation of riboflavin by singlet oxygen.

Aliquots of riboflavin [10 mM in 0.1 M acetate (Na^+, pH 4.5)] were incubated for up to 24 h in continuous light. Samples were taken at time-points and stored at –80ºC, then 20-µl aliquots were electrophoresed at pH 6.5 (**a**) or pH 2.0 (**b**). Orange G (internal marker) was circled in pencil, then the metabolites were stained with $AgNO_3$.
